# Efficacy and safety of ivermectin for the treatment of *Plasmodium falciparum* infections in asymptomatic male and female Gabonese adults – a pilot randomized, double-blind, placebo-controlled single-centre phase Ib/IIa clinical trial

**DOI:** 10.1016/j.ebiom.2023.104814

**Published:** 2023-10-13

**Authors:** Dorothea Ekoka Mbassi, Ghyslain Mombo-Ngoma, Jana Held, Dearie Glory Okwu, Wilfrid Ndzebe-Ndoumba, Laura Charlotte Kalkman, Franck Aurelien Ekoka Mbassi, Lais Pessanha de Carvalho, Juliana Inoue, Malik Azeez Akinosho, Lia Betty Dimessa Mbadinga, Emmanuel Koffi Yovo, Benjamin Mordmüller, Peter Gottfried Kremsner, Ayôla Akim Adegnika, Michael Ramharter, Rella Zoleko-Manego

**Affiliations:** a Centre de Recherches Médicales de Lambaréné, Lambaréné, Gabon; b Centre for Tropical Medicine, Bernhard Nocht Institute for Tropical Medicine & I. Department of Medicine, University Medical Center Hamburg-Eppendorf, Hamburg, Germany; c German Center for Infection Research (DZIF), Partner Site Hamburg-Borstel-Lübeck-Riems, Germany; d Department of Implementation Research, Bernhard Nocht Institute for Tropical Medicine & I. Department of Medicine, University Medical Center Hamburg-Eppendorf, Hamburg, Germany; e Institute of Tropical Medicine, University of Tübingen, Tübingen, Germany; f German Center for Infection Research (DZIF), Partner Site Tübingen, Tübingen, Germany; g Department of Medical Microbiology, Radboud University Medical Centre, Nijmegen, the Netherlands; h Department of Parasitology, Leiden University Medical Centre (LUMC), 2333 ZA, Leiden, the Netherlands; i Fondation pour la Recherche Scientifique, 72 BP45, Cotonou, Benin

**Keywords:** Ivermectin, *Plasmodium falciparum*, Gabonese adults, Clinical trial, Parasite reduction

## Abstract

**Background:**

Ivermectin's mosquitocidal effect and *in vitro* activity against *Plasmodium falciparum* asexual stages are known. Its *in vivo* blood-schizonticidal efficacy is unknown. Ivermectin's tolerability and efficacy against *P. falciparum* infections in Gabonese adults were assessed.

**Methods:**

The study consisted of a multiple dose stage and a randomized, double-blind, placebo-controlled stage. Adults with asymptomatic *P. falciparum* parasitaemia (200–5000 parasites/μl) were enrolled. First, three groups of five participants received 200 μg/kg ivermectin once daily for one, two, and three days, respectively, and then 34 participants were randomized to 300 μg/kg ivermectin or placebo once daily for 3 days. Primary efficacy outcome was time to 90% parasite reduction. Primary safety outcomes were drug-related serious and severe adverse events (Trial registration: PACTR201908520097051).

**Findings:**

Between June 2019 and October 2020, 49 participants were enrolled. Out of the 34 randomized participants, 29 (85%) completed the trial as per protocol. No severe or serious adverse events were observed. The median time to 90% parasite reduction was 24.1 vs. 32.0 h in the ivermectin and placebo groups, respectively (HR 1.38 [95% CI 0.64 to 2.97]).

**Interpretation:**

Ivermectin was well tolerated in doses up to 300 μg/kg once daily for three days and asymptomatic *P. falciparum* asexual parasitaemia was reduced similarly with this dose of ivermectin compared to placebo. Further studies are needed to evaluate plasmodicidal effect of ivermectin at higher doses and in larger samples.

**Funding:**

This study was funded by the Centre de Recherches Médicales de Lambaréné and the Centre for Tropical Medicine of the Bernhard Nocht Institute for Tropical Medicine.

**Research in context**

**Evidence before this study**

A search in PubMed on February 1, 2019, using the keywords “malaria”, “plasmodium”, “ivermectin”, “elimination”, “control”, “transmission”, and “safety” was performed that included English, French and German publications that were indexed in the online database with no limit on publication dates. Ivermectin is an established control tool against onchocerciasis and lymphatic filariasis. There is evidence for its mosquitocidal effect in malaria patients’ blood. Furthermore, *in vitro* models showed a plasmodicidal effect.

**Added value of this study**

This study evaluates the antimalarial effect and safety of ivermectin on *Plasmodium falciparum*, with the prospect to add that potential to the already established mosquitocidal effect. The good safety profile of ivermectin was confirmed. Ivermectin decreased the baseline parasitaemia below 90%. The same effect was observed in the control group taking placebo, failing to attribute the observed effect to ivermectin. These are new clinical results on the blood-schizonticidal effect of ivermectin.

**Implications of all the available evidence**

The data confirm that ivermectin remains a promising candidate to accompany other elimination tools due to its mosquitocidal effect. Participants achieved parasite reduction regardless of the intervention which should be re-evaluated in further clinical trials. Ivermectin administration as malaria treatment intervention should be evaluated at higher doses, with adequate study design.

## Introduction

Malaria remains a major threat to health and societal prosperity, especially in sub-Saharan African.^1^ Despite vector control, diagnosis, and treatment efforts, incidence of malaria remains high. Also, reduced activity of artemisinin-based combination therapies was reported.^2^ Therefore, new drugs with novel mechanisms of action are needed to improve malaria treatment.

Ivermectin is used on a large scale in mass drug administration (MDA) campaigns for the elimination of onchocerciasis and lymphatic filariasis since 1987.^3^ Ivermectin belongs to the avermectins, which cause the opening of invertebrate-specific glutamate-gated chloride channels resulting in flaccid paralysis and death.^4^ Its efficacy in killing Anopheles mosquitoes blood feeding from individuals who have received ivermectin is widely acknowledged, prompting its current pursuit for repurposing in MDA programs, to bolster efforts in controlling malaria vectors.^5^ Ivermectin is generally considered safe and well tolerated.^6^

Several studies showed that ivermectin MDAs at 150 μg/kg standard dose led to significant mortality in both wild-caught and laboratory-reared *Anopheles gambiae* mosquito populations for up to 14 days.^7^^,^^8^ While the recommended dose of ivermectin against lymphatic filariasis is up to 400 μg/kg, higher doses have been tested, e.g., in evaluations of the mosquitocidal activity, and showed acceptable safety.^6^^,^^9^ Malaria transmission can be reduced when persons have sufficient blood concentration of ivermectin as it kills mosquitoes after their blood meal.^10^ Modelling suggests that the main effect of ivermectin MDA is reduced size and life-expectancy of the total vector population (i.e., number of mosquitoes able to complete sporogony). And thus, ivermectin could reduce malaria incidence by 31–71% at the community level.^11^

*In vitro* studies suggest that ivermectin affects the life cycle of *Plasmodium falciparum* by inhibiting the nuclear import of polypeptides of the *P. falciparum* signal recognition particle (PfSRP). Both asexual and sexual stages have been evaluated.^12^^,^^13^ Ivermectin 400 μg/kg as a single dose had no effect on pre-erythrocytic stages of *P. falciparum* in a human challenge model.^14^ An *in vivo* blood-schizonticidal effect of ivermectin would have several benefits. The decrease in asexual parasitaemia would at the same time reduce the development of gametocytes and thus result in a prolonged transmission blocking effect of MDA interventions.^15^ Additionally, clearance of low parasitaemia could reduce or prevent anaemia and subclinical symptoms and prevent onset of symptomatic malaria.^16^ As ivermectin has a different mechanism of action than established antimalarial drugs, it would constitute an interesting combination partner in antimalarial chemotherapy.^17^

This study aimed to assess the safety of single doses of ivermectin 200 μg/kg for one, two, or three days and the blood-schizonticidal efficacy of ivermectin 300 μg/kg once daily for 3 days in adults with naturally acquired asymptomatic *P. falciparum* infection.

## Methods

### Study design and setting

This study was a seamless phase Ib/IIa, monocentric, sequential trial in adults with asymptomatic *P. falciparum* infection. The first stage was an open label, uncontrolled assessment of the safety of 200 μg/kg of ivermectin once daily for one, two and three days. The second stage was a randomized, double-blind, placebo-controlled assessment of the efficacy and safety of 300 μg/kg ivermectin once daily for three days (Supplementary Figure S1).

The study was conducted at the *Centre de Recherches Médicales de Lambaréné* (*CERMEL*)^18^ which is located in the Moyen-Ogooué province of Gabon. The region is endemic for malaria, showing a high prevalence of adult asymptomatic carriers of *P. falciparum*.^19^ The study protocol was registered with the Pan-African Clinical Trials Registry (PACTR201908520097051). There have been no major changes to trial outcomes after study commencement. The monitoring of safety data by a committee made of investigators and partners from the *Institute of Tropical Medicine*, Tübingen, Germany and *Bernhard Nocht Institute for Tropical Medicine*, Hamburg, Germany was performed after each set of five participants who received 200 μg/kg of ivermectin for one, two and three days, respectively, and completed their seven days of follow-up. The decision to continue to the subsequent group was based on the absence of grade 3 or serious treatment-related adverse events. That process led to the randomized-controlled stage.

### Participants

Potential participants, male and female, from Lambaréné and surrounding villages were pre-screened for *P. falciparum* infection by rapid diagnostic test, if positive followed by thick blood smear microscopy.

Adult participants (≥18 years) were eligible if they showed microscopic *P. falciparum* mono-infection of 200–5000 parasites/μl (P/μl), body weight ≥45 kg and body mass index (BMI) > 16.0 kg/m^2^, absence of fever (axillary temperature <37.5 °C and anamnestic absence of fever one week pre-inclusion), absence of signs and symptoms related to malaria, and willingness to comply to study procedures.

*Loa loa* is endemic in the study area^20^^,^^21^ and is a risk factor for serious neurological adverse events due to ivermectin administration in case of hypermicrofilaraemia.^22^ Therefore, individuals were not eligible in case of *L. loa* infection detected by thick blood smear microscopy upon screening. Other exclusion criteria included recent intake of antimalarial or ivermectin treatment, herbal medication, or experimental drugs, as well as intake of systemic antibiotics with known antimalarial activity, and no pregnancy nor breastfeeding (Supplementary Table S1).

### Enrolment, randomization, and blinding

During the first, multiple dose stage, eligible participants were consecutively enrolled in three groups of five participants each for the safety assessment of the study drug, no randomization or blinding was applied. Following the review of safety data at the end of each group by the safety review committee, recruitment into the subsequent group was opened. In the second stage of the study, participants were randomized to either ivermectin or placebo in an allocation ratio of 1:1 with 17 participants in each group. A study statistician independent of the clinical team and sponsor generated the random allocation sequence and randomization list prior to the study using a random number generator implemented in R Statistical Software (version 3.6.0; R Core Team 2019) with the package blockrand version 1.3 using random block sizes between 2 and 3. A pharmacy team, not involved in recruitment, patient care and outcome assessments, randomized patients according to the randomization list concealed in the pharmacy and oversaw study drug administration. Investigational team, participants, and microscopists were blinded to treatment administered to the participants and the randomization list was kept strictly confidential until final analysis.

### Intervention and procedures

During the multiple dose stage, participants received the standard dose of ivermectin 200 μg/kg once daily for one, two, or three days, respectively. In the randomized-controlled trial stage, participants received either ivermectin 300 μg/kg once daily for three days or placebo once daily for three days, based on dosage in a previous trial administering ivermectin to *Plasmodium*-infected participants.^23^ The number of tablets for placebo-treated participants equalled the number of tablets for ivermectin-treated participants with identical body weight. Ivermectin and placebo were administered orally with water after intake of butter croissant. Tablets were concealed in neutral plastic bags so that participants were not able to distinguish ivermectin from placebo. Drug administration was done in a separate room by a pharmacy team member to maintain double blinding. The first dose was administered directly after successful eligibility assessment. Subsequent doses were given at the same time point ±30 min on the following two days, when applicable.

Placebo tablets were Winthrop's *P-Tabletten Weiss* 7 mm *Lichtenstein,* which do not contain any active ingredient. They were purchased at the University Pharmacy Tübingen, Germany. Ivermectin tablets were MSD's *Stromectol* 3 mg, purchased in Gabon. No quality analysis was performed on the drug batches used.

At enrolment, participants underwent physical examination with demographic data and medical history recorded. Blood samples were taken for haematology (red blood cell count, haemoglobin, haematocrit, leukocyte count with differential and platelet count), clinical chemistry (AST, ALT, urea, creatinine, CRP, and electrolytes), urinalysis, thick and thin blood smears, and for quantitative polymerase chain reaction (qPCR). Blood pregnancy tests were performed at baseline for female participants. Participants were hospitalized for 72 h. Following discharge from the research centre, participants returned for outpatient visits at days 4, 5, 6, and 7. Participants received rescue medication with artemether-lumefantrine (Novartis Coartem® 80/480) in case of fever (axillary temperature >38.5 °C) in conjunction with any *Plasmodium* parasitaemia or upon development of danger signs, or severe malaria, or parasitaemia ≥20,000 parasites/μl. All other participants were treated for malaria on day 7. Safety assessments were performed from first drug administration until end of study. These included daily physical examination, vital signs, and inquiry about adverse events (AEs), as well as clinical laboratory tests (haematology, clinical chemistry, urinalysis) upon screening and days 3, 5, and 7. AEs were followed up until resolution.

### Determination of parasitaemia by microscopy

Blood smears (two thick and thin smears) were prepared at baseline, hour (H) 8, H16, H24, H32, H40, H48, H56, H64, H72, and at days 4, 5, 6, and 7. The smears were dried and stained with either 10% Giemsa stain (Cat: 1.09204, Sigma–Aldrich) for 15 min for screening smears or with 3% Giemsa stain for 60 min for all other timepoints. Each stained smear was read by Lambaréné method^24^ by two independent microscopists qualified for the detection of asexual and sexual *Plasmodium* parasites. A smear was considered negative if no asexual parasites or gametocytes were detected in 200 high-power fields instead of the usual 100 fields. Asexual parasite density, expressed as parasites per μl (P/μl) of blood, was calculated by averaging the two counts. Microscopy results were considered non-concordant in case of difference of parasitaemia of ≥50% for parasitaemia ≥300P/μl, difference of parasitaemia of ≥100P/μl for parasitaemia <300P/μl, difference in positivity, or difference in *Plasmodium* species determination. Non-concordance was resolved by a third microscopist.

### Quantitative polymerase chain reaction (qPCR)

Samples for parasite quantification and genotyping by qPCR were collected at the same time points as blood smears. Whole blood was stabilized with RNA*later*™ (Cat: AM7021, Thermo Fisher Scientific) and later analysed. Total nucleic acid (DNA and RNA) was purified from these samples using the QIAamp DNA blood mini kit (Cat: 51106, Qiagen) according to the manufacturer's instructions and the sbeadex blood kit (Cat: NAP44410, LGC) automated in the KingFisher™ Flex Purification System (Thermo Fisher Scientific). Purified nucleic acid samples were kept at −20 °C until use. Reverse-transcription quantitative polymerase chain reaction (RT-qPCR) was carried out targeting a conserved region of the 18s RNA gene of *Plasmodium spp.* Reactions were performed using TaqMan® RNA-to-Ct™ 1-Step Kit (Cat: 4392938, Thermo Fisher Scientific) and primers (Integrated DNA Technologies) and probe (Eurofins Genomics) previously published^25^^,^^26^ with a minor modification in the probe: 5′ HEX fluorophore and 3′ Eclipse Quencher were attached to the minor groove binder molecule. Samples were assayed in duplicates along with no-template and positive controls on the LightCycler 480 Instrument II (Roche Applied Science). The crossing point (Cp) values were calculated with the second derivative maximum method (LightCycler 480 software: version 1.5.1.62). *P. falciparum* laboratory strain 3d7 (MR-102) synchronized at ring stage and counted by microscopy were serially diluted to generate a standard curve used for calculation of the samples’ parasitaemia.

### Outcomes

The primary efficacy endpoint was the time to 90% reduction of asexual parasitaemia, consistent for at least 8 h, assessed by microscopy. Secondary efficacy endpoints were the time to 90% reduction of asexual parasitaemia assessed by qPCR, the parasite clearance time, defined as time to parasitaemia <100P/μl by qPCR, and the difference in AUC of parasitaemia until day 7.

The primary safety endpoint was the number of treatment-emergent serious adverse events (SAEs) and severe adverse events (AEs) from the time of first administration of study drugs until day 7. The secondary safety endpoint was the number of any AE from the time of first administration of study drugs up to day 7. AE relationship to the study medication was assessed by the investigators and classified as unrelated, unlikely, possibly, probably, and definitely related. AE intensity was categorized as mild, moderate, or severe.

To address the potential issue of multiplicity of outcomes, the priorities of the endpoints were specified in advance in the study protocol and statistical analysis plan. No post-hoc outcomes were added and results from secondary outcomes served to support the confirmatory results from the primary outcome.

### Sample size

The multiple dose stage served as safety assessment and did not have any sample size calculation, an empiric number of five participants per group was chosen. The sample size of the randomized-controlled trial stage was calculated with the assumption that 25% of participants allocated to placebo would reduce parasitaemia to 90% of the initial value within 7 days due to naturally acquired immunity, while at least 75% of ivermectin-treated participants would reduce parasitaemia by 90%. Considering a power of 90%, a single-sided α of 2.5% and a ratio of 1:1 (ivermectin vs. placebo), 17 participants per group were required.

### Statistical analysis

Data were transcribed from paper case report forms to the REDCap electronic data capture system version 8.3.1. hosted at CERMEL. Analyses were done using Stata IC version 17.0 (StataCorp LLC, College Station, Texas, USA) with the package Table 1_mc.^27^

**Table 1:** Baseline characteristics of the intention-to-treat population (n = 49).

a) Multiple dose stage				
Group	1d-200 μg/kg IVM (n = 5)	2d-200 μg/kg IVM (n = 5)	3d-200 μg/kg IVM (n = 5)	Total (n = 15)
Age, years	24.0 (20.0–39.0)	20.0 (19.0–37.0)	32.0 (27.0–59.0)	27.0 (19.0–49.0)
Sex				
Male	3 (60%)	3 (60%)	2 (40%)	8 (53%)
Female	2 (40%)	2 (40%)	3 (60%)	7 (47%)
Weight, kg	51.0 (46.0–55.0)	63.4 (53.0–71.0)	64.0 (59.0–71.0)	57.0 (51.0–71.0)
Height, cm	167.0 (164.0–171.0)	167.0 (156.0–168.0)	158.0 (151.0–166.0)	166.0 (155.0–170.0)
Body-mass index, kg/m^2^	19.1 (19.0–19.5)	25.2 (17.6–26.4)	26.1 (25.6–28.5)	20.4 (19.0–26.4)
Axillary body temperature, °C	36.5 (35.6–36.5)	36.4 (36.0–36.6)	36.4 (36.0–36.7)	36.4 (36.0–36.7)
Parasitaemia, parasites/μl	295 (277–352)	503 (314–863)	317 (298–599)	317 (277–618)

b) Randomized-controlled trial stage			
Group	3d-300 μg/kg IVM (n = 17)	3 d-placebo (n = 17)	Total (n = 34)
Age, years	25.0 (21.0–33.0)	24.0 (19.0–40.0)	25.0 (20.0–40.0)
Sex			
Male	10 (59%)	13 (76%)	23 (68%)
Female	7 (41%)	4 (24%)	11 (32%)
Weight, kg	62.0 (57.0–69.0)	62.0 (58.9–65.0)	62.0 (58.0–69.0)
Height, cm	161.0 (153.0–168.0)	168.0 (163.0–173.0)	165.5 (158.0–172.0)
Body-mass index, kg/m^2^	21.7 (21.3–24.4)	22.8 (20.5–25.2)	22.0 (20.7–25.2)
Axillary body temperature, °C	36.5 (36.0–36.7)	36.6 (36.2–36.7)	36.5 (36.1–36.7)
Parasitaemia, parasites/μl	426 (268–677)	1102 (619–2413)	663 (426–1529)

Data are median (interquartile range) and n (%), IVM: ivermectin.

All participants who received at least one dose of study treatment were considered the intention-to-treat population (ITT) and the safety population was made of everyone enrolled in the study. The per-protocol population (PP population) consisted of all participants who received all doses designated to the allocated group, had a mono-infection with *P. falciparum*, as assessed by microscopy, and completed follow-up until day 7. Descriptive statistics are presented as numbers and proportions for categorical variables and median and interquartile range for quantitative variables.

The seven days follow-up parasitaemia in the ivermectin and placebo groups was compared using the Kaplan–Meier method for survival analysis. Time to event was defined as the time of 90% parasite reduction, otherwise, participants were censored at the day last seen. Median survival time was given with 95% confidence intervals, wherever the variability of the sample allowed. Groups were compared using log-rank test and Cox regression for point estimate and 95% confidence intervals. A post-hoc Wilcoxon Breslow test was performed on advice by the reviewer. The same analysis was done in both ITT and PP populations.

For the qPCR parasite reduction and parasite clearance time (asexual parasite load <100 P/μl), the same analyses as with the primary outcome were performed.

AUC of log-10 transformed parasitaemia until day 7 has been calculated by trapezoidal rule and compared between ivermectin and placebo group by Student's t-test. Parasitaemia values have been augmented by a constant of half the limit of detection before log-transformation.^28^, ^29^, ^30^

A two-sided α < 5% was used as a statistical significance threshold and statistics are presented with a p value as well as a confidence interval where appropriate. All statistical test results are tabulated.

Adverse events were recorded from the first drug administration until the end of active follow-up at day 7. Verbatim-recorded AEs have been coded using MedDRA version 3.0, The proportion of participants with AEs, and the proportion of AEs, classified by preferred term level, were tabulated. Time until onset of dermatological AEs has been analysed post-hoc.

### Ethical considerations

The study was approved by the Institutional Ethics Committee of *CERMEL* (CEI/CERMEL 006/2019) on 16 May 2019. The study was conducted according to the ICH-GCP and the declaration of Helsinki as well as all applicable national laws and guidelines. All participants signed a written informed consent before any study-specific procedure was initiated.

It was retrospectively registered with the Pan African Clinical Trials Registry (PACTR201908520097051) on 18 July 2019 due to technical difficulties. At the time of IEC approval, the trial registry was not available due to maintenance and connection issues. In order to meet both the requirement to make study information transparently available and logistical constraints to start recruiting, the study was registered in MESA Track on 28 May 2019^31^ before opening of recruitment on 05 June 2019. As visible in the MESA Track and trial registration, as well as statistical analysis plan and recruitment log available upon request, there have been no changes to outcomes or endpoints of the study at any time and at successful trial registration, only 5 out of 49 study participants had been enrolled.

### Role of the funding source

The sponsor of the study was *CERMEL*. The trial was financed using institutional funds of *CERMEL* and the *Department of Clinical Research* of the *Bernhard Nocht Institute for Tropical Medicine*. Study design, data collection, analysis, interpretation and writing of the report, as well as the decision to submit the paper for publication were done solely by the authors.

## Results

### Participants recruitment and flow

**Fig. 1:**
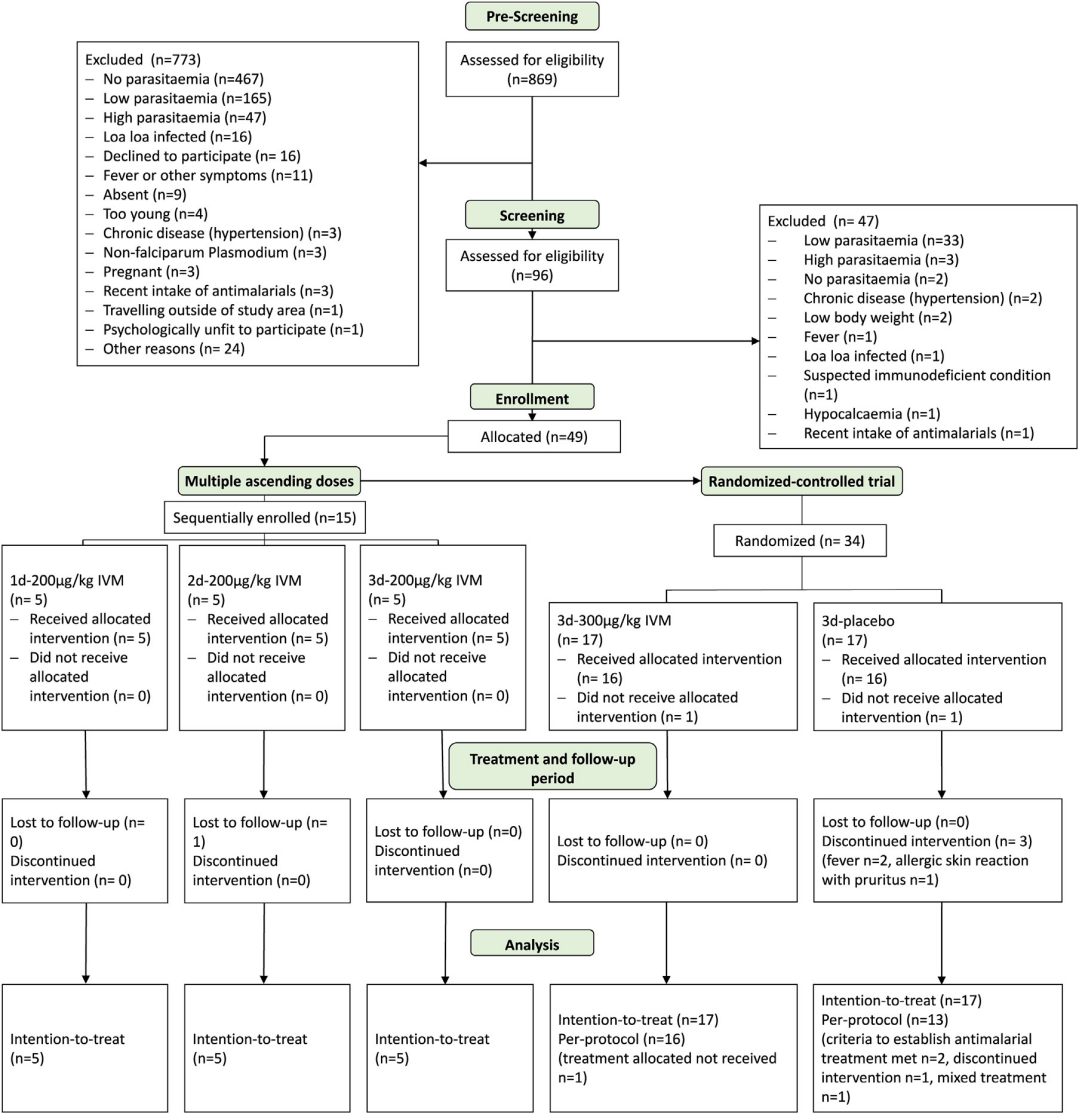
**Participant flow and numbers analysed in intention-to-treat and per-protocol population**.

Participants recruitment and follow-up were conducted between 05 June 2019 and 07 October 2020. Out of 869 volunteers pre-screened for malaria, 96 with positive malaria parasite counts were invited to participate and sign an informed consent form. Following screening, 49 eligible volunteers were consecutively enrolled in the study. Reasons for ineligibility were mainly parasitaemia below 200 P/μl (72%, Fig. 1). All 49 participants enrolled in the study were included in the ITT and safety populations. Two participants in the 1d-200 μg/kg IVM were shown not to fulfil all eligibility criteria at verification after treatment administration. One had a parasitaemia ≤200 P/μl, one had a BMI <16.0 kg/m^2^. Of the 34 randomized participants, one on 3d-300 μg/kg ivermectin was excluded from PP analysis because of not having received the allocated treatment and four participants on 3 d-placebo were excluded. One of them received mixed treatment, one discontinued the intervention because of skin rash and pruritus, and two participants were excluded because they received rescue treatment (Fig. 1). Although not a PP population exclusion criterion, of note is that species detection in qPCR revealed that only 20/49 (41%) participants were mono-infected with *P. falciparum* throughout follow-up. Non-falciparum (*malariae, ovale*) *Plasmodium* was detected in at least one sample of 28/49 (57%) participants, 1/49 (2%) participant was shown to have a *P. malariae* monoinfection.

### Participants’ baseline characteristics

Overall, considering the ITT population, the median age was 25 (IQR 20–40) years with 25 (21–33) in the 3d-300 μg/kg ivermectin group and 24 (19–40) in the 3 d-placebo group (Table 1). Globally, there were slightly more male than female participants, even in the randomized stage (53% vs. 47% during first, 68% vs. 32% during second stage). All participants were Black Africans. Median body mass index was 20.4 (19.0–26.4) kg/m^2^ and 22.0 (20.7–25.2) kg/m^2^ in the respective trial stages. All participants were afebrile at enrolment with a median axillary body temperature of 36.4 (36.0–36.7) °C, and 36.5 (36.1–36.7) °C in first and second stage, respectively. The median *P. falciparum* parasitaemia was 317 (277–618) P/μl in the multiple dose stage, and 663 (426–1529) P/μl in the randomized-controlled stage. It was similar across the different study groups except in the placebo group where it tended to be higher. An ad-hoc analysis showed that baseline characteristics between ITT and PP populations were similar (Supplementary Table S2).

### 90% Parasite reduction

**Fig. 2:**
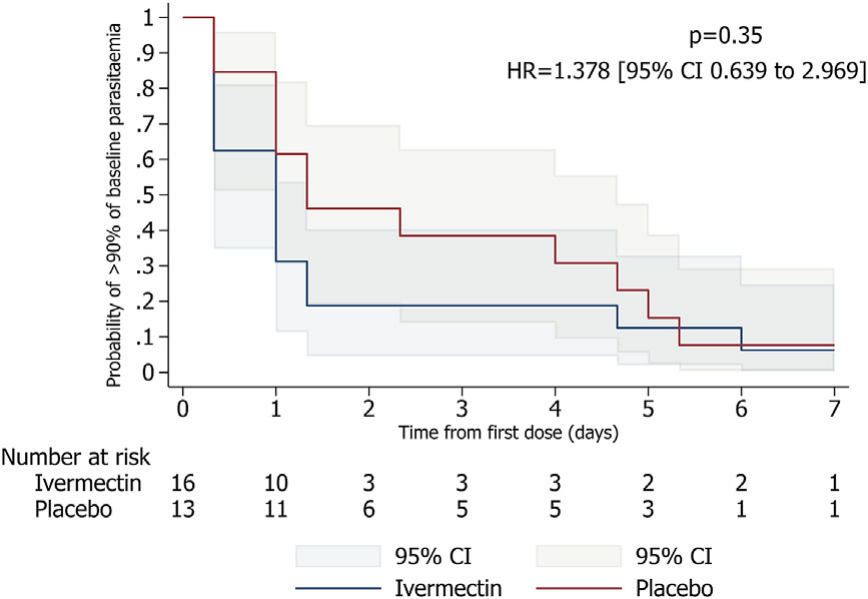
**Kaplan–Meier curve for the randomized-controlled trial stage in per-protocol population (n** = **29):** Time to >90% parasite reduction by thick blood smear, p value (log-rank test) and hazard ratio (Cox regression).

15/16 (94%) and 12/13 (92%) of participants on 3d-300 μg/kg ivermectin and placebo, respectively, reached 90% parasite reduction of asexual parasitaemia within a median time of 24.07 [95% CI 8.03–31.83] hours vs. 32.0 [95% CI 24.03–115.60] hours, respectively. 3d-300 μg/kg ivermectin compared to placebo showed a hazard of 90% parasite reduction 38% higher in the intervention group though the evidence was not strong (hazard ratio (HR) = 1.38 [95% CI 0.64–2.97], Fig. 2). Comparable results were observed by intention-to-treat analysis (Supplementary Figure S2, parasite reduction median time 24.07 [95% CI 8.03–32.02] hours vs. 32.00 [95% CI 24.05–115.60] hours, Table 2).

**Table 2:** Efficacy results for the randomized-controlled trial stage.

a) Survival analyses				
	p value (log-rank test)	p value (Wilcoxon–Breslow–Gehan test)	Hazard ratio (Cox regression)	95% Confidence interval (Cox regression)
**Parasite reduction**				
Per-protocol population (n = 29)				
By thick blood smear	0.350	0.123	1.378	0.639–2.969
By qPCR	0.388	0.731	0.670	0.255–1.759
Intention-to-treat population (n = 34)				
By thick blood smear	0.477	0.095	1.267	0.606–2.648
By qPCR	0.514	0.878	0.756	0.311–1.837
**Parasite clearance**				
Per-protocol population by qPCR (n = 29)	0.767	0.338	1.123	0.484–2.607
Intention-to-treat population by qPCR (n = 34)	0.836	0.340	1.079	0.484–2.407

b) Area under the curve				
By per-protocol population (n = 29)	t statistic (Student's t-test)	p value (Student's t-test)	Mean of difference (Student's t-test)	95% Confidence interval (Student's t-test)
By thick blood smear	−0.532	0.599	−13.222	−64.217 to 37.774
By qPCR	−0.109	0.914	−3.125	−61.820 to 55.571

The qPCR 90% parasite reduction outcome showed that 3d-300 μg/kg ivermectin could be associated with 33% lower hazard of parasite reduction compared to placebo, though with no robust evidence (HR = .67 [95% CI 0.26–1.76], Supplementary Figure S3). The median parasite reduction time was 141.38 h vs. 94.12 h in the ivermectin and placebo groups, respectively. Results were not different in the intention-to-treat population (Supplementary Figure S4, ivermectin 141.38 vs. placebo 94.12 h). Post-hoc analyses showed not statistically significantly different results (Table 2).

### Parasite clearance <100P/μl

The parasite clearance time of asexual *P. falciparum* developmental stages, defined as time to <100P/μl by qPCR, was assessed. In the per-protocol population of the randomized-controlled trial stage, median parasite clearance time was similar at 32.02 [95% CI 8.10–76.00] vs. 56.03 [95% CI 32.00–124.00] hours and a HR of 1.12 [95% CI 0.48–2.61] (Supplementary Figure S5, Table 2). In the intention-to-treat population, parasite clearance was not statistically significantly different between randomized groups either (Supplementary Figure S6, Table 2, parasite clearance time ivermectin 32.00 [95% CI 14.02–76.00] vs. placebo 40.03 [95% CI 32.00–56.05] hours).

### Difference in parasite area under the curve

**Fig. 3:**
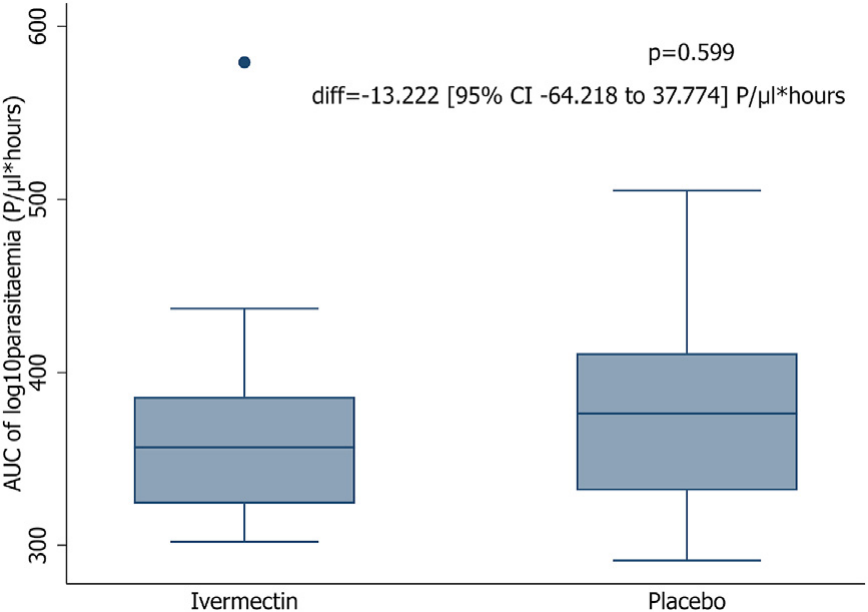
**Area under the curve of log10-transformed parasitaemia in per-protocol population (n** = **29):** for the randomized-controlled stage by thick blood smear, p value and mean difference (Student's t-test).

There was no significant difference in parasite AUC between ivermectin and placebo either in thick blood smear- or in qPCR-derived parasitaemia of the PP population during the randomized-controlled trial stage (Fig. 3, p = 0.60, mean difference −13.22 [95% CI −64.22 to 37.77] P/μl∗hours and Supplementary Figure S7, p = 0.91, mean difference −3.13 [95% CI −61.82 to 55.57] P/μl∗hours).

### Adverse events

No severe or serious AEs were reported during the trial. Overall, 29/49 (59%) enrolled participants experienced at least one transient mild or moderate AEs.

The number of participants experiencing AEs and frequency of AEs increased with number of doses during multiple dose stage. Furthermore, during the randomized-controlled trial stage, possibly or probably related AEs were more frequent in the 3d-300 μg/kg ivermectin group (8/11 [73%]) than in the placebo group (3/11 [27%], Table 3). Most AEs possibly or probably related to study treatment were allergic dermatitis (4/6 [67%] for multiple dose stage and 5/11 [45%] for randomized controlled trial stage), pruritus (1/8 [13%] and 3/11 [27%], respectively) and urticaria (1/8 [13%] and 2/11 [18%], respectively). During the randomized-controlled trial stage, dermatological AEs appeared after the second dose (4/7 [57%] for 3d-300 μg ivermectin vs. 3/3 [100%)] for 3 d-placebo) or third dose (3/7 [43%] for 3d-300 μg ivermectin vs. 0 for 3 d-placebo).

**Table 3:** All adverse events from study drug administration in the intention-to-treat population (n = 49).

a) Multiple dose stage				
Group	1d-200 μg/kg IVM	2d-200 μg/kg IVM	3d-200 μg/kg IVM	Total
All participants	n = 5	n = 5	n = 5	n = 15
Patients with at least 1 AE	2 (40.00)	4 (80.00)	5 (100.0)	11 (73.33)
Possibly or probably treatment-related AEs	n = 2	n = 1	n = 5	n = 8
Allergic dermatitis	1 (50.00)	0	2 (40.00)	3 (37.50)
Pruritus	0	0	1 (20.00)	1 (12.50)
Urticaria	0	0	1 (20.00)	1 (12.50)
Diarrhoea	0	0	1 (20.00)	1 (12.50)
Headache	0	1 (100.00)	0	1 (12.50)
Mouth ulceration	1 (50.00)	0	0	1 (12.50)
Dermatological AEs	n = 1	n = 1	n = 4	n = 6
Allergic dermatitis	1 (100.00)	1 (100.00)	2 (50.00)	4 (66.67)
Pruritus	0	0	1 (25.00)	1 (16.67)
Urticaria	0	0	1 (25.00)	1 (16.67)
All AEs	n = 3	n = 6	n = 12	n = 21
Headache	1 (33.33)	2 (33.33)	5 (41.67)	8 (38.10)
Allergic dermatitis	1 (33.33)	1 (16.67)	2 (16.67)	4 (19.05)
Pyrexia	0	0	2 (16.67)	2 (9.52)
Urticaria	0	0	1 (8.33)	1 (4.76)
Diarrhoea	0	0	1 (8.33)	1 (4.76)
Pruritus	0	0	1 (8.33)	1 (4.76)
Anaemia	0	1 (16.67)	0	1 (4.76)
Palpitations	0	1 (16.67)	0	1 (4.76)
Oropharyngeal pain	0	1 (16.67)	0	1 (4.76)
Mouth ulceration	1 (33.33)	0	0	1 (4.76)

b) Randomized-controlled trial stage			
Group	3d-300 μg/kg IVM	3 d-placebo	Total
All participants	n = 17	n = 17	n = 34
Patients with at least 1 AE	10 (58.82)	8 (47.06)	18 (52.94)
Possibly or probably treatment-related AEs	n = 8	n = 3	n = 11
Allergic dermatitis	4 (50.00)	1 (33.33)	5 (45.45)
Pruritus	1 (12.50)	2 (66.67)	3 (27.27)
Urticaria	2 (25.00)	0	2 (18.18)
Constipation	1 (12.50)	0	1 (9.09)
Dermatological AEs	n = 7	n = 3	n = 10
Allergic dermatitis	4 (57.14)	1 (33.33)	5 (50.00)
Pruritus	1 (14.29)	2 (66.67)	3 (30.00)
Urticaria	2 (28.57)	0	2 (20.00)
All AEs	n = 16	n = 13	n = 29
Headache	4 (25.00)	1 (7.69)	5 (17.24)
Allergic dermatitis	4 (25.00)	1 (7.69)	5 (17.24)
Pyrexia	0	3 (18.75)	3 (9.38)
Pruritus	1 (6.25)	2 (12.50)	3 (9.38)
Myalgia	1 (6.25)	1 (7.69)	2 (6.90)
Urticaria	2 (12.50)	0	2 (6.90)
Anaemia	0	1 (7.69)	1 (3.45)
Arthralgia	0	1 (7.69)	1 (3.45)
Chills	0	1 (7.69)	1 (3.45)
Conjunctivitis	0	1 (7.69)	1 (3.45)
Fatigue	0	1 (7.69)	1 (3.45)
Constipation	1 (6.25)	0	1 (3.45)
Urinary tract infection	1 (6.25)	0	1 (3.45)
Nausea	1 (6.25)	0	1 (3.45)
Periorbital oedema	1 (6.25)	0	1 (3.45)

AEs are given as preferred term (PT), data are n (%), IVM: ivermectin.

Study treatment was discontinued in one participant in the placebo group due to allergic dermatitis. Overall, four participants received early antimalarial treatment before day 7, one on 2d-200 μg ivermectin and three in the placebo group. None of the participants on 3d-300 μg/kg ivermectin met criteria for early antimalarial treatment.

## Discussion

Ivermectin is given for mass drug administration to whole communities to control lymphatic filariasis and onchocerciasis. It has been observed in some studies that ivermectin can kill *Anopheles* when they feed on the blood of people who have taken this medication. The assumption is therefore that mass drug administration of ivermectin to whole communities will lead to the killing of many *Anopheles* mosquitoes and could reduce malaria transmission.^32^ While some studies have been conducted at the community level^33^, ^34^, ^35^, ^36^ to assess the impact of ivermectin on malaria, the present study aimed to assess ivermectin as a treatment for malaria parasite reduction at the individual level.

Within seven days of follow-up, more than 90% participants reduced parasite load by 90% in both ivermectin and placebo groups. The time to achieve 90% parasite reduction was 24 h vs. 32 h in ivermectin and placebo groups, respectively. However, this was not statistically significant as confidence intervals overlapped. The finding of similar reduction of parasite count between the intervention and control group suggests that in the study population, 90% parasite reduction within seven days could be achieved without use of ivermectin. The 90% parasite reduction in placebo group is likely driven by the fact that the study participants were semi-immune adults. This therefore challenges our sample size calculation assumption that only 25% of participants in the placebo group would achieve 90% parasite reduction. Nonetheless, there are indications that parasite reduction might be quicker in the ivermectin (24 h) than in the placebo group (32 h). Confidence intervals of the median time to 90% parasite reduction for ivermectin range from 8 h to 32 h while in the placebo group they range from 24 h to more than 115 h suggesting a trend for ivermectin. The hazard ratio of 1.38 is also in favour of an effect of ivermectin above that of placebo. There was statistically no evidence to support the superiority of ivermectin from either per protocol or ITT analyses suggesting there was no attrition bias that could affect that assessment. Also, it cannot be ruled out that the slightly slower parasite reduction in the placebo group might be due to the higher baseline parasitaemia. No subgroup analysis was deemed necessary because of the small sample size, the lack of statistically significant differences between the randomized groups and because a recent PK study that showed no association between ivermectin pharmacokinetics and sex, or BMI.^8^ Still, disparities were observed with overall more men than women, and the higher baseline parasitaemia in the control group despite randomization.

The secondary outcomes analyses support the primary outcome results as there are some trends that the parasite clearance time is shorter in the ivermectin group compared to the placebo group. The same is shown for the area under the curve of parasitaemia that is smaller for ivermectin. However, all these findings do not show strong statistical evidence that would comfort any conclusion and rule out the role of chance.

A clear differentiation between asymptomatic and pre-symptomatic participants cannot be made. Therefore, some participants developed malaria symptoms only hours after inclusion in the trial. Indeed, semi-immunity that is characterized by the host's ability to tolerate and, to an extent, supress blood stage infection, is assumed to be acquired over the course of several disease episodes.^37^^,^^38^ It can therefore be questioned whether the choice of a semi-immune asymptomatic population for assessment of efficacy is justified. On the other hand, it would have been unethical to evaluate ivermectin in non-immune *P. falciparum*-infected subjects and therefore the choice of a semi-immune study population was correct.

In a clinical study investigating the efficacy of ivermectin against onchocerciasis before the drug was licensed (1985–1987), the lack of efficacy of ivermectin against *Plasmodia* was already briefly mentioned. However, this was only a sub-analysis and no tangible numbers were presented, whereas the present study was specifically designed to provide evidence on the effect of ivermectin on *Plasmodium falciparum*.^39^

The stringent application of a randomized, placebo-controlled clinical trial design supports the solidity of the presented results. The drop-out rate was low with only one participant lost to follow-up and three participants having discontinued the intervention. None of the participants in the trial had missing outcome data. As microscopy was the primary diagnostic tool, these results should be easily reproducible across research centres in malaria-endemic areas.

The findings on safety showed a good tolerability of ivermectin at the doses of 200 μg/kg once daily up to three days and 300 μg/kg once daily for three days. This is in line with reports of higher doses showing that ivermectin is well tolerated in humans up to 2000 μg/kg.^40^, ^41^, ^42^, ^43^ Beside the fact that ivermectin dose is not the driver of *Loa loa* treatment-related encephalopathy, the approach in this study was very cautious based on scientific review committee advice. Firstly, anyone with microscopically detectable L. loa was excluded, and secondly, safety for the standard dose of 200 μg/kg of ivermectin single dose was evaluated, then increased to two and three days, before moving to a very conservative dose of 300 μg/kg for three days for the efficacy assessment. The exclusion of subjects with *L. loa* microfilaremia detectable has eliminated the risk for *L. loa* treatment-related encephalopathy.^22^ The concomitant administration of study medication with fatty food contrasts with the cautious measures taken as it is described that this increases the bioavailability of ivermectin^43^ and could therefore expose the participants to a higher and potentially unsafe dose. Our safety outcomes suggest no complications.

There are a few limitations to this study. These include a small error in the sample size calculation as it occurs after post-hoc checks that with the assumptions made, the number per arm should have been approximately 16, 19 to be exact, while it was set to 17. Nonetheless, that mistake is believed not to significantly affect the findings providing the assumptions were correct. However, the assumption to have 25% and 75% achieving the outcome in the ivermectin and placebo groups, respectively, was not confirmed as more than 90% reduced the parasite load up to 90% in both arms. This suggest that the sample size could have been underpowered and thus the study was too small to detect the real effect. There is a need to adjust power and sample size calculation for future trials. Considering our observed hazard ratio of 1.378 as well as the rate of parasite reduction in the placebo group were true, setting conventional 5% α and 80% power, a sample size of 260 participants would be needed. However, as discussed, our sample size hypothesis and endpoint might not have been adequate. Our findings and findings from other studies on asymptomatic volunteers will be helpful in better defining the variability of parasites in this population, thus providing more robust hypothesises for power and sample size calculation of trials investigating blood-schizonticidal effect of ivermectin.

The single centre study design limits the external validity and thus the generalization of the findings. This is an early phase, proof of concept study, so internal validity might be most important at this stage.

As no quality analysis was performed on the drug batches used, it cannot be ruled out that the absence of drug effect may have been due to sub-standard drug quality. Placebo and ivermectin were sourced from good manufacturing practice-certified manufacturers in batches produced for commercial delivery. Having procured these drugs from accredited pharmacies, we assume they have not been sub-standard.

This is an in-human trial evaluating the efficacy of ivermectin against *Plasmodium spp*. blood stage infection. It was successful in informing future studies. The intervention was well tolerated with no severe adverse events observed. At the evaluated dose, asymptomatic *P. falciparum* asexual parasitaemia was reduced regardless of the treatment group. A dose-dependent effect of ivermectin on *Plasmodium* cannot be ruled out, as the effective dose may likely be higher. Further studies large in sample size and adequately designed are needed to further characterise plasmodicidal effect of ivermectin.

## Contributors

JH, MR and GMN conceived the study. LP and RZM drafted the protocol. AAA, PGK, BM, DEM, GMN, JH, MR and RZM contributed to the refinement of the protocol and approved the final version. DEM, DGO, EKY, GMN, LBDM, LCK, MAA, RZM and WNN conducted the investigations. DEM and FAEM did the fieldwork. JI performed parasitological analyses. BM was the trial statistician. DEM and EKY were responsible for data curation. DEM, RZM and BM accessed and verified the data. BM, DEM and GMN analysed the data. DEM wrote the first draft of the report with input from GMN, MR and RZM. All authors read and approved the final manuscript before submission.

## Data sharing statement

The anonymized data that support the findings of this study, its protocol, and related documents, are available with publication for access via the *Centre de Recherches Médicales de Lambaréné* (cermel.org). Requests for access will be reviewed by a Data Access Committee to ensure that use of data protects the interests of the participants and researchers according to the terms of ethics approval and principles of equitable data sharing. Requests can be submitted by email to bertrand.lell@cermel.org via the Data Access Form available at cermel.org/accessing-data. The CERMEL platform is registered with the Registry of Research Data Repositories (re3data.org).

## Declaration of interests

BM's institution has recently received a grant by *Deutsches Zentrum für Infektionsforschung (DZIF)* that is not related to this manuscript. He is a DSMB member for the MultiMal study (PACTR202008909968293), for which he is not remunerated. All other authors declare no competing interests.

## Appendix A Supplementary data

Supplementary Table S2

Supplementary Figure S1 **Study design:** seamless phase Ib/IIa sequential trial with multiple dose stage and randomized-controlled trial stage.

Supplementary Figure S2 **Kaplan-Meier curve for the randomized-controlled trial stage in intention-to-treat population (n=34):** Time to >90% parasite reduction by thick blood smear, p value (log-rank test) and hazard ratio (Cox regression).

Supplementary Figure S3 **Kaplan-Meier curve for the randomized-controlled trial stage in per-protocol population (n=29):** Time to >90% parasite reduction by qPCR, p value (log-rank test) and hazard ratio (Cox regression).

Supplementary Figure S4 **Kaplan-Meier curve for the randomized-controlled trial stage in intention-to-treat population (n=34):** Time to >90% parasite reduction by qPCR, p value (log-rank test) and hazard ratio (Cox regression).

Supplementary Figure S5 **Kaplan-Meier curve for the randomized-controlled trial stage in per-protocol population (n=29):** Time to parasite clearance by qPCR, p value (log-rank test) and hazard ratio (Cox regression).

Supplementary Figure S6 **Kaplan-Meier curve for the randomized-controlled trial stage in intention-to-treat population (n=34):** Time to parasite clearance by qPCR, p value (log-rank test) and hazard ratio (Cox regression).

Supplementary Figure S7 **Area under the curve of log10-transformed parasitaemia in per-protocol population (n=29):** for the randomized-controlled stage by qPCR, p value and mean difference (Student’s t-test).

Synopsis IVERCURE MESA TRACK PROTOCOL VERSION 3

DSMB charter

Protocol

Statistical Analysis Plan
