# Supplementary material for: Efficacy and safety of ivermectin for the treatment of Plasmodium falciparum infections in asymptomatic male and female Gabonese adults – a pilot randomized, double-blind, placebo-controlled single-centre phase Ib/IIa clinical trial
Source: eBioMedicine. 2023 Oct 13;97:104814. doi: 10.1016/j.ebiom.2023.104814 (PMC10582777; doi:10.1016/j.ebiom.2023.104814)
Supplement: Supplementary Table S1 [file mmc1.docx]

*Supplementary table 1.* ***Eligibility criteria as per trial protocol***

| **Inclusion criteria** |
| --- |
| Male or female, aged ≥ 18 years and body weight ≥ 45 kg |
| *P. falciparum* parasitaemia of 200 to 5000 parasites/μL |
| Asymptomatic *P. falciparum* infection defined as: presence of *P. falciparum* mono-infection with absence of fever (axillary temperature <38.5 °C and absence of history of fever in recent 24 hours and the week before inclusion) and other symptoms related to malaria. |
| Willingness to take part in the study and to sign the informed consent form |
| **Exclusion criteria** |
| Active tuberculosis, or history of taking anti-tuberculosis medications within 12 months prior to screening |
| any *Loa loa microfilaria* infection detected by microscopy |
| AST/ALT > 2x the upper limit of normal range (ULN) |
| Taking an experimental drug in the last 4 weeks |
| Antimalarial treatment in the last 4 weeks |
| Use of systemic antibiotics with known antimalarial activity within 30 days of study enrolment (e.g., trimethoprim-sulfamethoxazole, doxycycline, tetracycline, clindamycin, erythromycin, fluoroquinolones, or azithromycin). |
| Use of ivermectin within 30 days of study enrolment |
| Participants taking herbal medication within one week of screening |
| Known or suspected electrolyte imbalance, e.g., hypokalaemia, hypocalcaemia or hypo-magnesemia with clinical significance |
| Moderate to severe anaemia (Haemoglobin level <8 g/dL) |
| Any confirmed or suspected immunosuppressive or immunodeficient condition, including human immunodeficiency virus (HIV) infection |
| Severe malnutrition (Body Mass Index (BMI) < 16.0) |
| Pregnant or nursing (lactating) women |
| Known chronic underlying disease such as sickle cell disease or severe cardiac impairment |
| Participants with serum creatinine ≥ 2 X ULN in the absence of dehydration. In case of dehydration, Participants with serum creatinine ≥ 2 X ULN after oral or parenteral re-hydration |
| Participants with any psychiatric or neurological condition including substance abuse |
| Allergy to ivermectin |
