# Supplementary material for: Efficacy and safety of ivermectin for the treatment of Plasmodium falciparum infections in asymptomatic male and female Gabonese adults – a pilot randomized, double-blind, placebo-controlled single-centre phase Ib/IIa clinical trial
Source: eBioMedicine. 2023 Oct 13;97:104814. doi: 10.1016/j.ebiom.2023.104814 (PMC10582777; doi:10.1016/j.ebiom.2023.104814)
Supplement: Supplementary Table S2 [file mmc2.docx]

*Supplementary table 2.* ***Baseline characteristics of the per-protocol population (n=38).*** *Data are median (interquartile range) and n (%), IVM: ivermectin.*

1. Multiple dose stage

| **group** | **1d-200µg/kg IVM (n=2)** | **2d-200µg/kg IVM (n=4)** | **3d-200µg/kg IVM (n=3)** | **total (n=9)** |
| --- | --- | --- | --- | --- |
| age, years | 29·5 (20·0-39·0) | 19·5 (18·5-28·5) | 32·0 (27·0-64·0) | 27·0 (20·0-37·0) |
| sex  male  female | 1 (50%)  1 (50%) | 3 (75%)  1 (25%) | 1 (33%)  2 (67%) | 5 (56%)  4 (44%) |
| weight, kg | 53·0 (51·0-55·0) | 62·0 (51·0-73·5) | 71·0 (55·0-72·0) | 55·0 (53·0-71·0) |
| height, cm | 165·5 (164·0-167·0) | 167·5 (161·5-172·0) | 151·0 (139·0-166·0) | 166·0 (156·0-167·0) |
| body-mass index, kg/m^2^ | 19·4 (19·0-19·7) | 21·4 (17·4-28·2) | 28·5 (26·1-31·1) | 25·2 (19·0-28·5) |
| axillary body temperature, °C | 36·5 (36·5-36·5) | 36·3 (36·0-36·7) | 36·4 (34·9-37·2) | 36·5 (36·0-36·6) |
| parasitaemia, parasites/µl | 324 (295-352) | 589 (281-2712) | 317 (298-754) | 317 (298-754) |

1. Randomized-controlled trial stage

| **group** | **3d-300µg/kg IVM (n=16)** | **3d-placebo (n=13)** | **total (n=29)** |
| --- | --- | --- | --- |
| age, years | 25·5 (21·0-37·5) | 22·0 (19·0-25·0) | 24·0 (20·0-30·0) |
| sex  male  female | 9 (56%)  7 (44%) | 9 (69%)  4 (31%) | 18 (62%)  11 (38%) |
| weight, kg | 63·2 (58·0-69·5) | 61·0 (58·9-65·0) | 62·0 (58·9-69·0) |
| height, cm | 163·0 (153·0-169·0) | 168·0 (161·0-175·0) | 165·0 (157·0-172·0) |
| body-mass index, kg/m^2^ | 22·0 (21·4-25·4) | 22·8 (19·3-25·2) | 22·3 (20·9-25·2) |
| axillary body temperature, °C | 36·5 (36·0-36·7) | 36·5 (36·2-36·7) | 36·5 (36·1-36·7) |
| parasitaemia, parasites/µl | 448 (273-871) | 844 (619-2378) | 648 (426-1104) |
