# Supplementary figures and images for: Efficacy and safety of ivermectin for the treatment of Plasmodium falciparum infections in asymptomatic male and female Gabonese adults – a pilot randomized, double-blind, placebo-controlled single-centre phase Ib/IIa clinical trial
Source: eBioMedicine. 2023 Oct 13;97:104814. doi: 10.1016/j.ebiom.2023.104814 (PMC10582777; doi:10.1016/j.ebiom.2023.104814)

## Multiple ascending dose stage

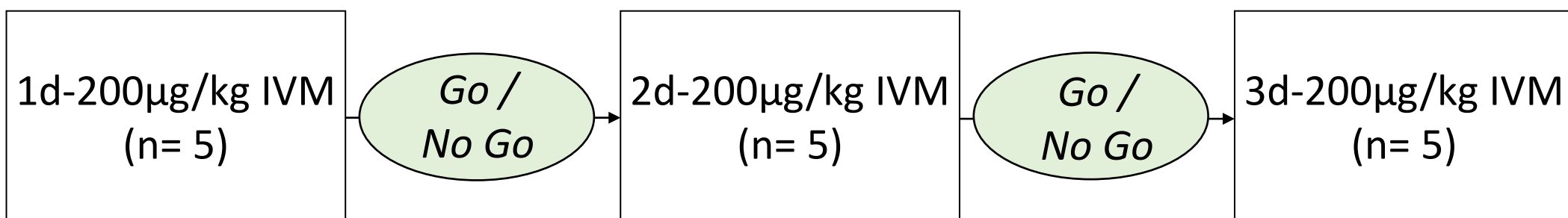

## Randomized-controlled trial stage

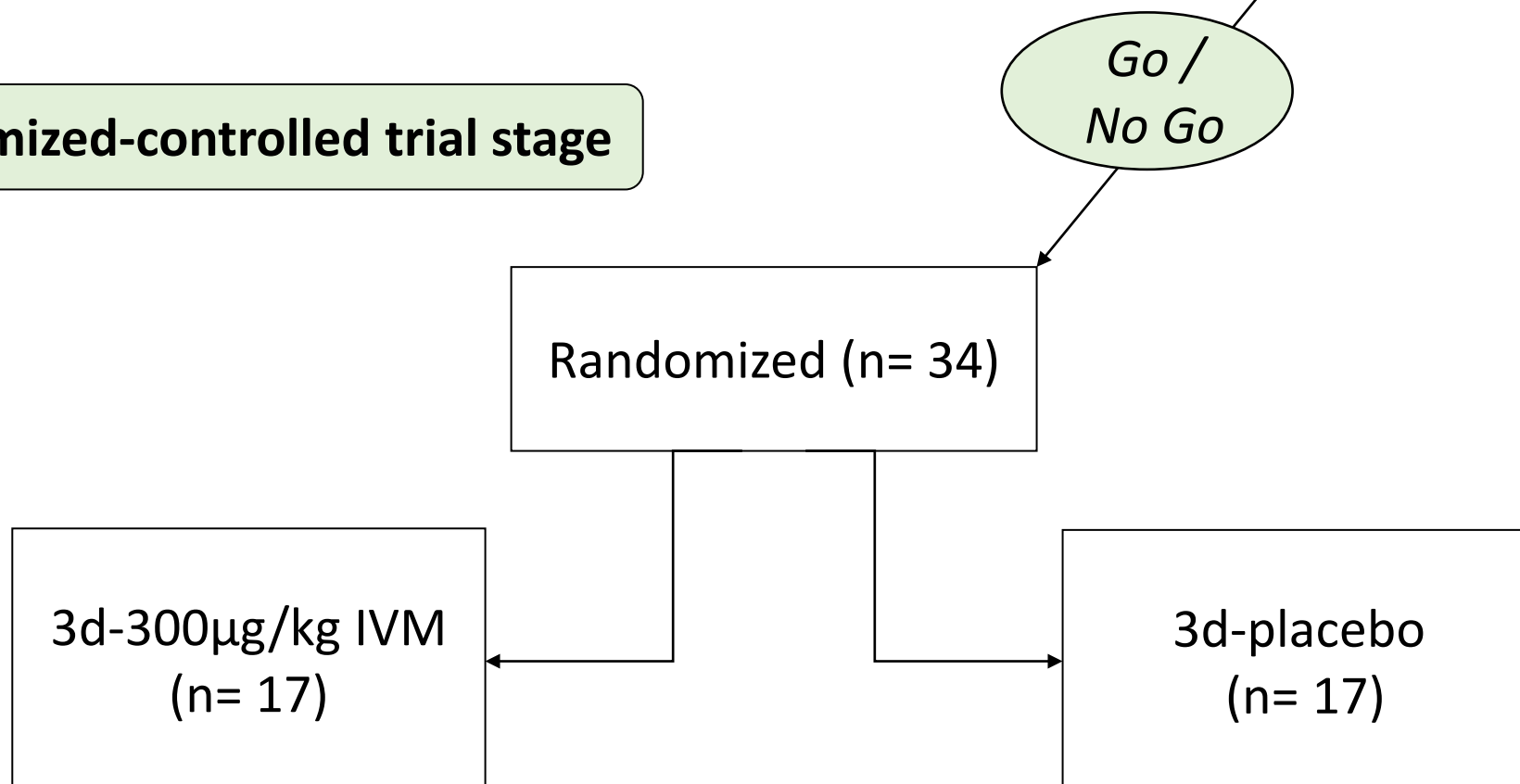

Supplement: Supplementary Figure S1 — Study design: seamless phase Ib/IIa sequential trial with multiple dose stage and randomized-controlled trial stage. [file mmc3.pdf]

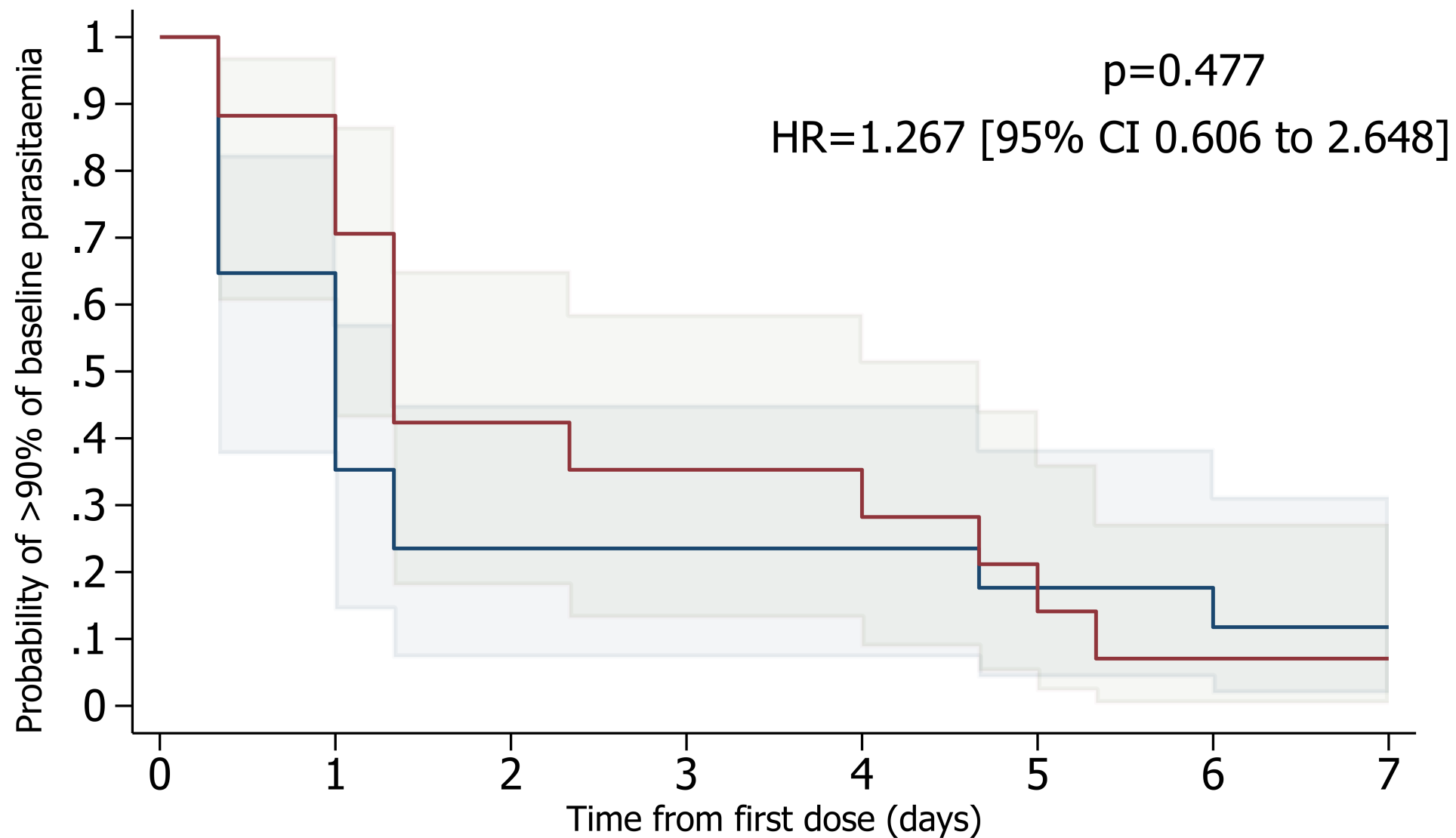

Number at risk

Ivermectin 17 11 4 4 4 3 3 2

Placebo 17 15 6 5 5 3 1 1

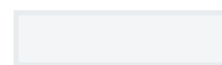

95% CI

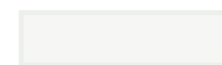

95% CI

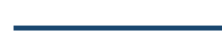

Ivermectin

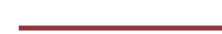

Placebo

Supplement: Supplementary Figure S2 — Kaplan-Meier curve for the randomized-controlled trial stage in intention-to-treat population (n=34): Time to >90% parasite reduction by thick blood smear, p value (log-rank test) and hazard ratio (Cox regression). [file mmc4.pdf]

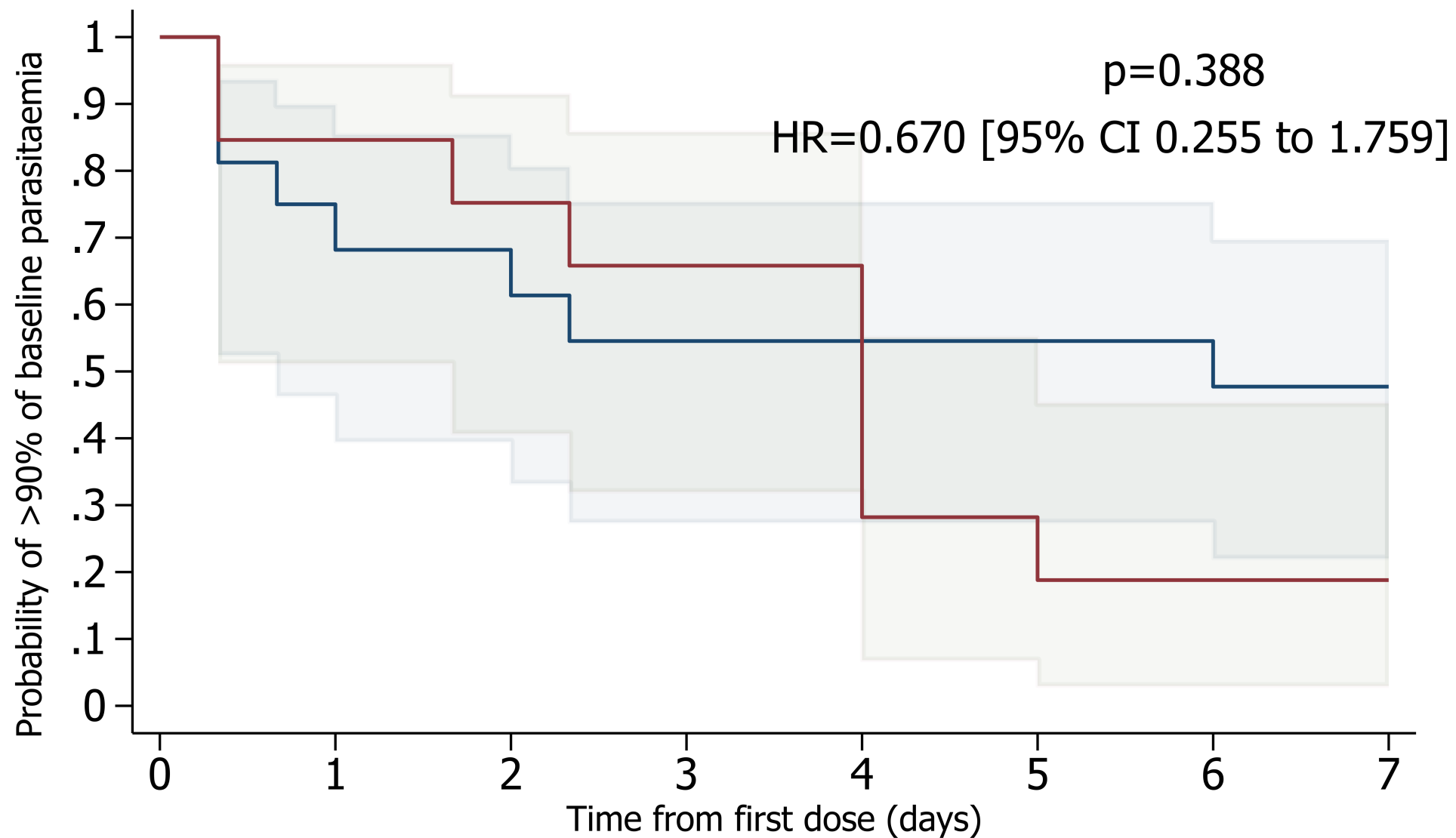

Number at risk

|            |    |    |    |   |   |   |   |   |
|------------|----|----|----|---|---|---|---|---|
| Ivermectin | 16 | 11 | 10 | 8 | 8 | 8 | 8 | 6 |
| Placebo    | 13 | 9  | 8  | 7 | 7 | 3 | 2 | 2 |

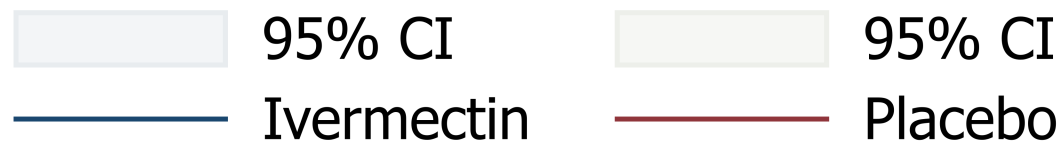

Supplement: Supplementary Figure S3 — Kaplan-Meier curve for the randomized-controlled trial stage in per-protocol population (n=29): Time to >90% parasite reduction by qPCR, p value (log-rank test) and hazard ratio (Cox regression). [file mmc5.pdf]

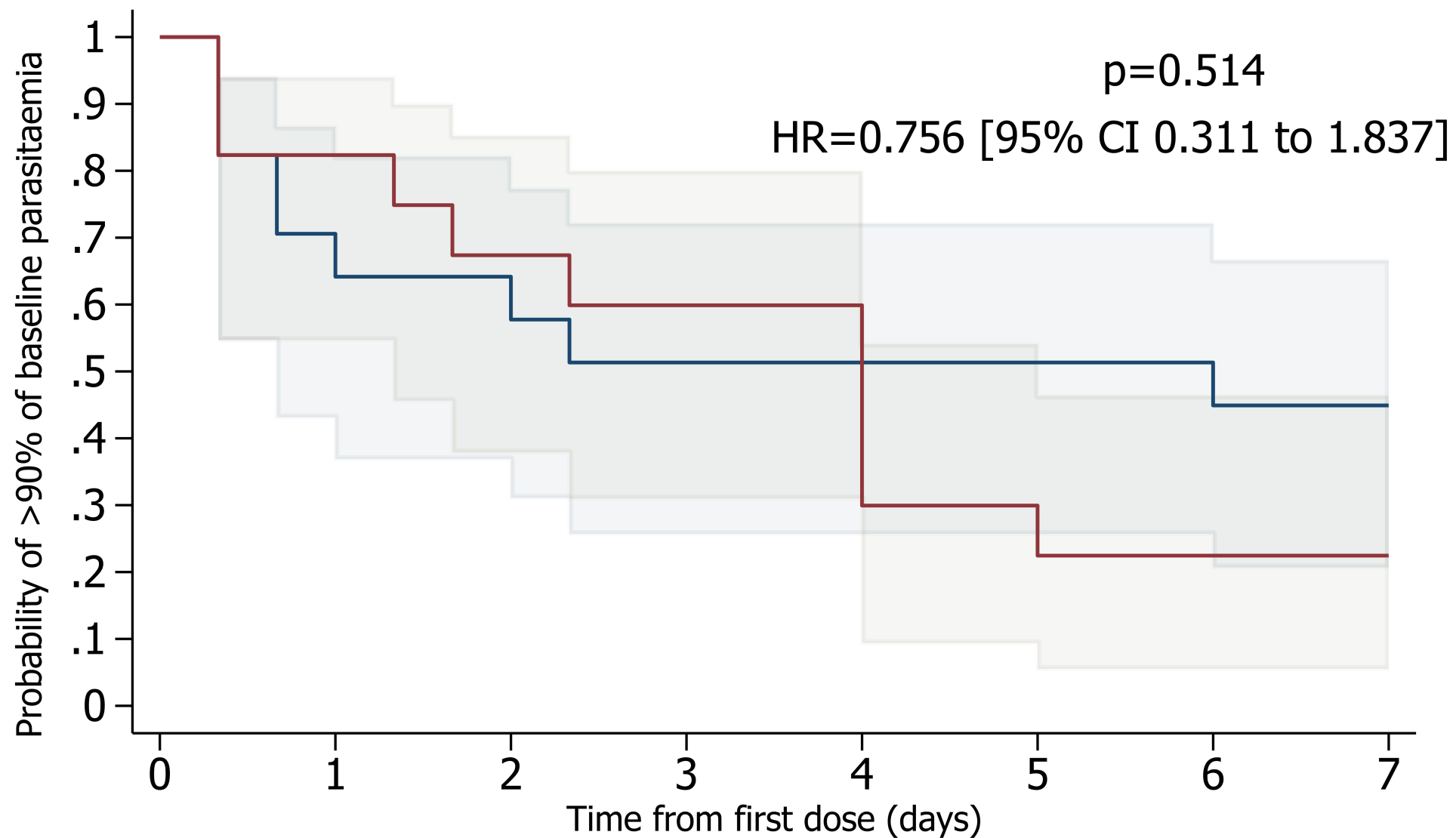

Number at risk

|            |    |    |    |   |   |   |   |   |
|------------|----|----|----|---|---|---|---|---|
| Ivermectin | 17 | 11 | 10 | 8 | 8 | 8 | 8 | 6 |
| Placebo    | 17 | 12 | 9  | 8 | 8 | 4 | 3 | 3 |

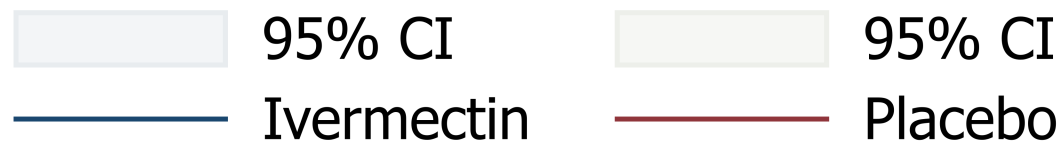

Supplement: Supplementary Figure S4 — Kaplan-Meier curve for the randomized-controlled trial stage in intention-to-treat population (n=34): Time to >90% parasite reduction by qPCR, p value (log-rank test) and hazard ratio (Cox regression). [file mmc6.pdf]

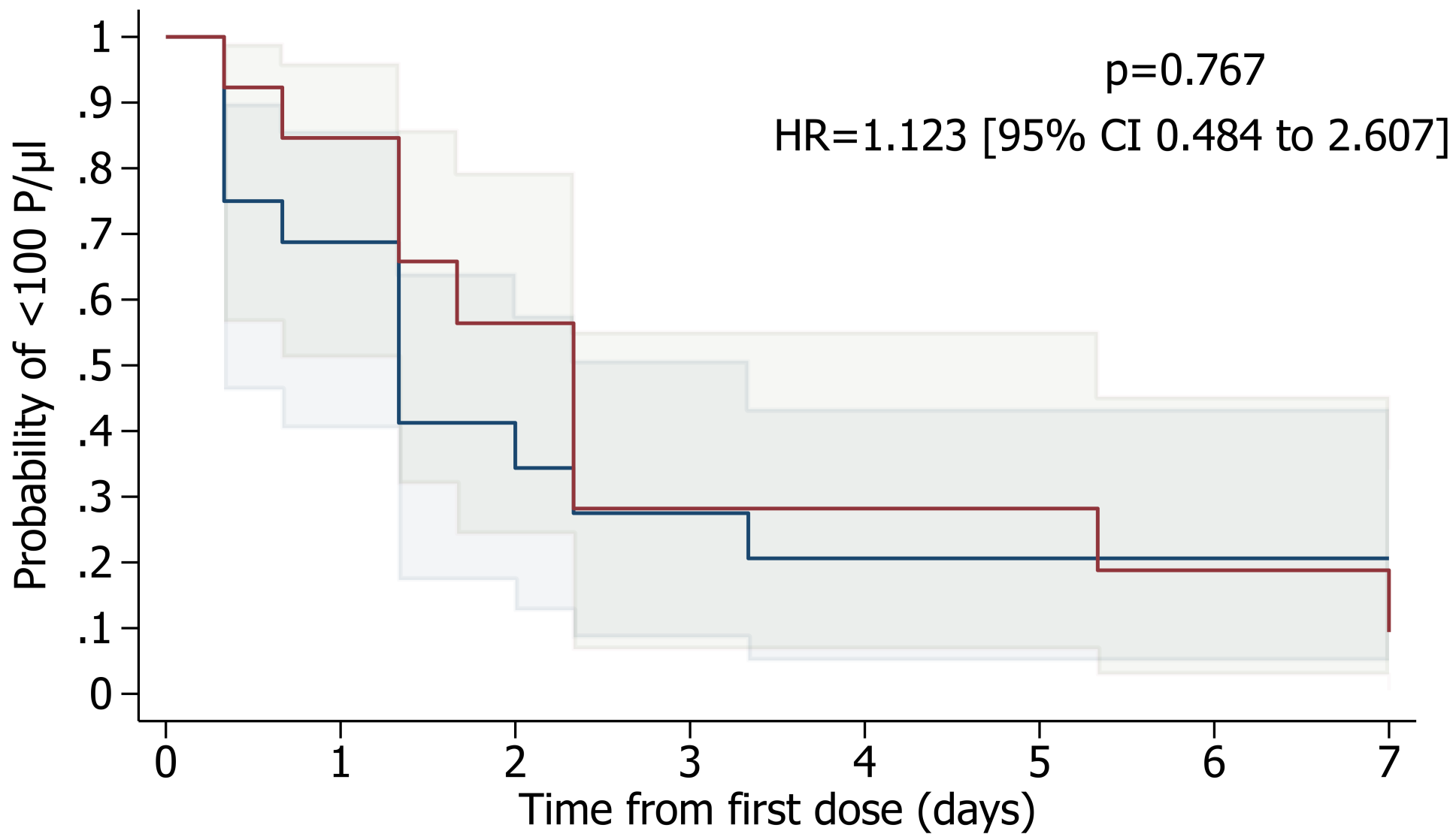

Number at risk

|            |    |    |   |   |   |   |   |   |
|------------|----|----|---|---|---|---|---|---|
| Ivermectin | 16 | 10 | 6 | 4 | 3 | 3 | 3 | 3 |
| Placebo    | 13 | 9  | 6 | 3 | 3 | 3 | 2 | 2 |

Supplement: Supplementary Figure S5 — Kaplan-Meier curve for the randomized-controlled trial stage in per-protocol population (n=29): Time to parasite clearance by qPCR, p value (log-rank test) and hazard ratio (Cox regression). [file mmc7.pdf]

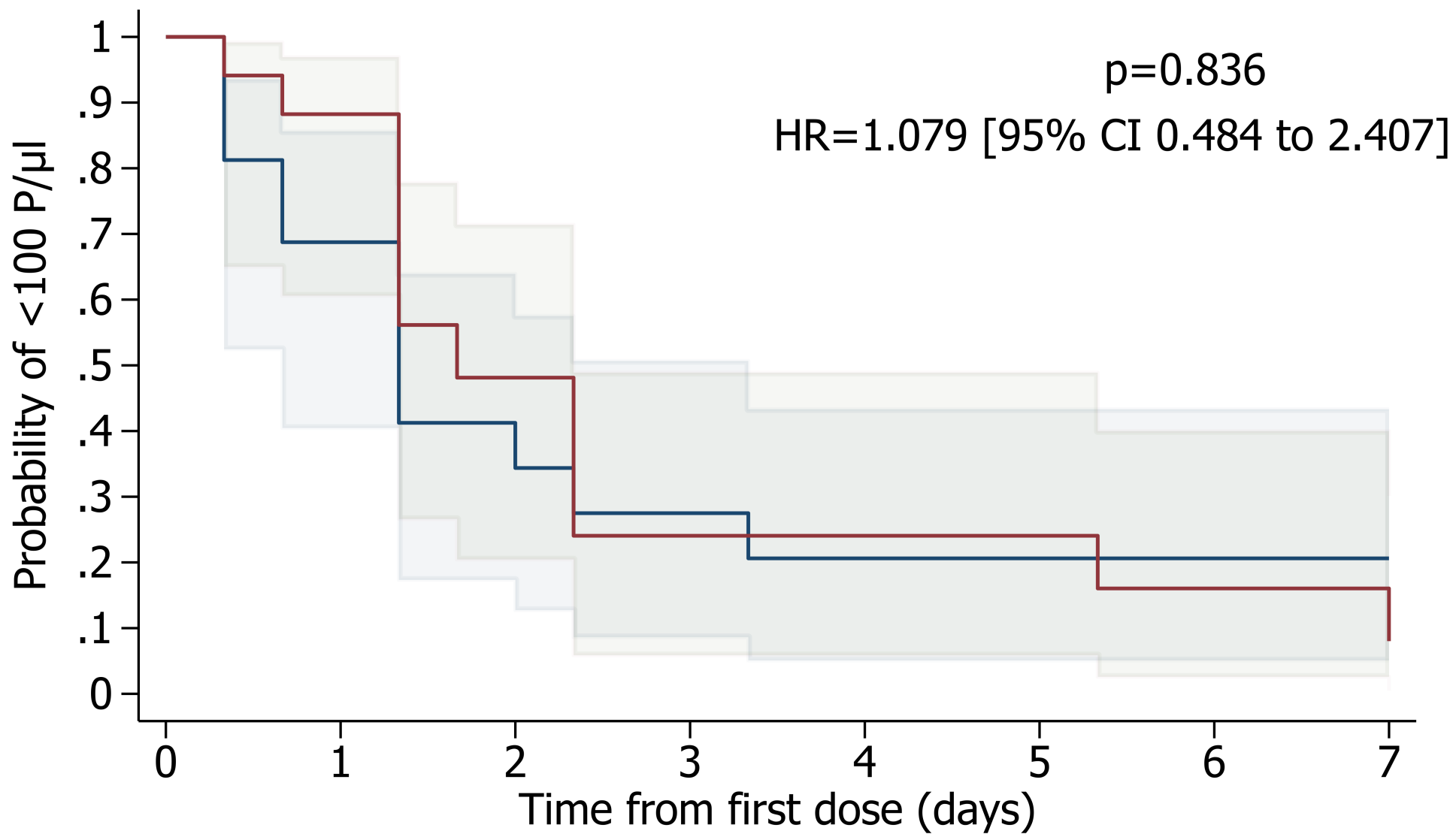

Number at risk

|            |    |    |   |   |   |   |   |   |
|------------|----|----|---|---|---|---|---|---|
| Ivermectin | 16 | 10 | 6 | 4 | 3 | 3 | 3 | 3 |
| Placebo    | 17 | 13 | 6 | 3 | 3 | 3 | 2 | 2 |

95% CI  
Ivermectin  
95% CI  
Placebo

Supplement: Supplementary Figure S6 — Kaplan-Meier curve for the randomized-controlled trial stage in intention-to-treat population (n=34): Time to parasite clearance by qPCR, p value (log-rank test) and hazard ratio (Cox regression). [file mmc8.pdf]

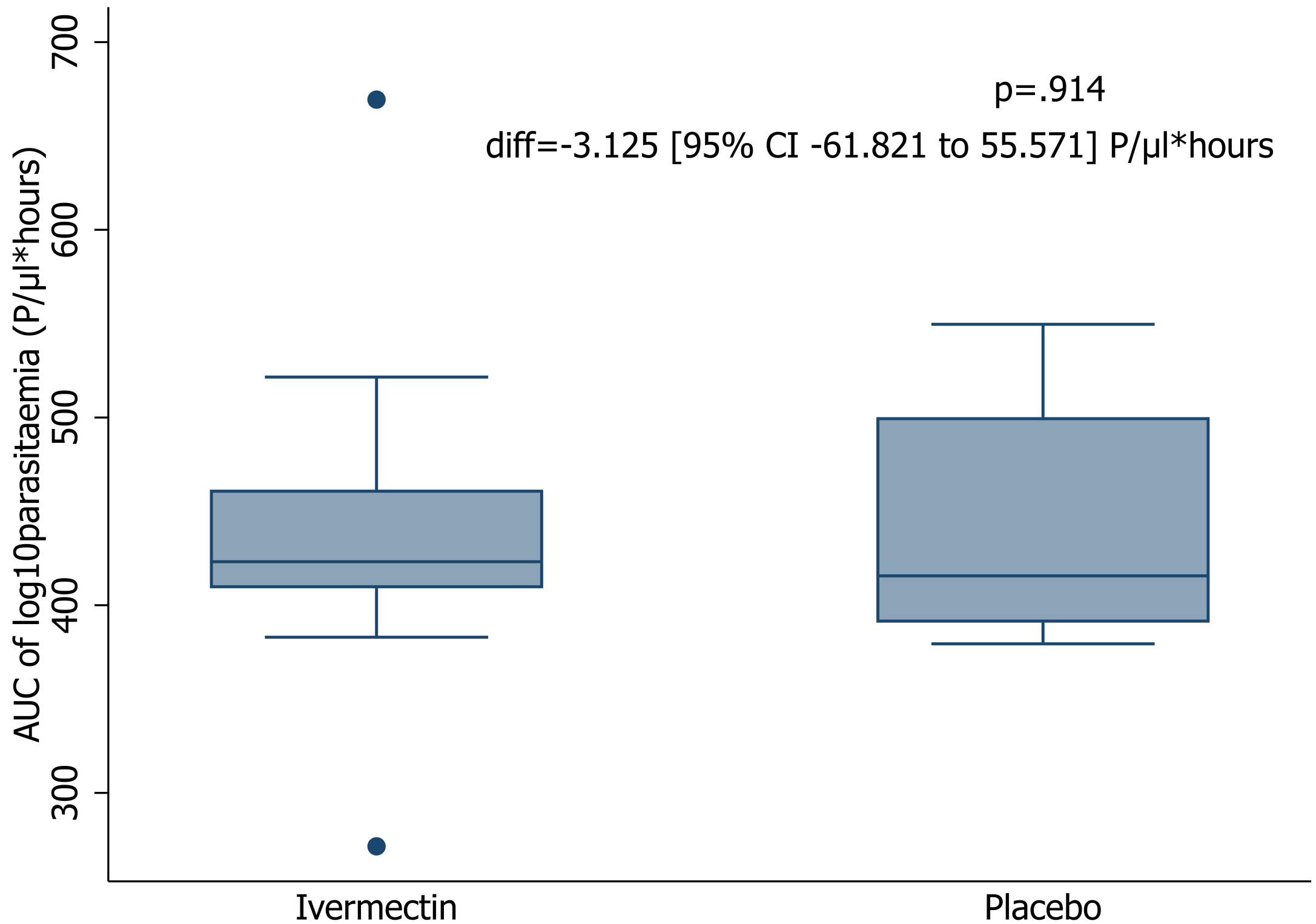

Supplement: Supplementary Figure S7 — Area under the curve of log10-transformed parasitaemia in per-protocol population (n=29): for the randomized-controlled stage by qPCR, p value and mean difference (Student’s t-test). [file mmc9.pdf]
