# Supplementary material for: Efficacy and safety of ivermectin for the treatment of Plasmodium falciparum infections in asymptomatic male and female Gabonese adults – a pilot randomized, double-blind, placebo-controlled single-centre phase Ib/IIa clinical trial
Source: eBioMedicine. 2023 Oct 13;97:104814. doi: 10.1016/j.ebiom.2023.104814 (PMC10582777; doi:10.1016/j.ebiom.2023.104814)
Supplement: Synopsis IVERCURE MESA TRACK PROTOCOL VERSION 3 [file mmc10.pdf]

Clinical Trial Synopsis

Efficacy and safety of Ivermectin for the treatment of *Plasmodium falciparum* infections in asymptomatic Gabonese adults

Protocol version 3 (30-04-2019)

Trial Sponsor:	Centre de Recherches Médicales de Lambaréné (CERMEL)
Investigator:	Rella Zoleko-Manego, MD, MSc
Coordinating Investigator:	Jana Held, PhD
Co-Investigator:	Ghyslain Mombo-Ngoma, MD, PhD
Authors:	Lais Carvalho
	Dorothea Sträßner
	Wilfrid Ndoumba, MD
	Lia Betty Dimessa, MD
	Malick Akinosho, MD
	Mirjam Groger, MD, PhD
	Sebastian Wicha
Statistician:	Benjamin Mordmüller, MD, PhD
Safety Monitor:	Michael Ramharter, MD, MSc, DTMH

**Study title**	Efficacy and safety of Ivermectin for the treatment of *Plasmodium falciparum* infections in asymptomatic Gabonese adults
**Study acronym**	**IVERCURE**
**Protocol version**	V.3
**Protocol date**	30 April 2019
**Clinical Phase**	1
**Trial Centre(s)**	CERMEL, Gabon
**Rationale**	Ivermectin has a potent anti-parasitic and anti-insecticide activity against many organisms including ecto- and endoparasites in animals and in humans.<br><br>Malaria, a mosquito-borne disease caused by parasites of the genus *Plasmodium*, remains the most important parasitic disease in humans worldwide. It has been shown that ivermectin can reduce transmission of *Plasmodium* parasites by its activity against blood-sucking mosquitoes, but recent *in vitro* data suggests that ivermectin has also and effect on the erythrocytic stages of *P. falciparum*, <u>but *in vivo* data are lacking.</u><br><br>Besides its insecticidal activity, the excellent safety profile of ivermectin, which has been used for decades in mass drug administration programs, and a different mechanism of action compared to other antimalarials make ivermectin an interesting candidate for malaria control and elimination campaigns.<br><br>This dose escalation study is designed to assess the activity of ivermectin on the parasitaemia of asymptomatic *P. falciparum* infections. The aim of this study is to investigate whether ivermectin is safe at a 3x300 µg/kg treatment course and can reduce or clear parasitaemia in participants infected with *P. falciparum* assessed by thick blood smear microscopy.
**Primary Objective(s)**	- To assess the safety and tolerability of single- and multiple ascending doses of ivermectin in volunteers with asymptomatic *P. falciparum* infection<br>- To assess the efficacy of ivermectin in participants with asymptomatic *P. falciparum* infection
**Secondary Objective(s)**	- To compare efficacy of ivermectin 200µg/kg single dose versus two-day 200µg/kg treatment versus three-day 200µg/kg treatment versus three-day 300µg/kg treatment

	- To compare efficacy of ivermectin three-day 300µg/kg treatment compared to placebo<br>- To compare safety and tolerability of ivermectin 200µg/kg single dose versus two-day 200µg/kg treatment versus three-day 200µg/kg treatment versus three-day 300µg/kg treatment<br>- To compare safety and tolerability of ivermectin three-day 300µg/kg treatment compared to placebo
**Exploratory Objectives**	To assess:<br>- Effect of pharmacokinetic parameters on efficacy on safety<br>- Activity of ivermectin on *P. falciparum* gametocytes<br>- Parasite kinetics in the placebo group<br>- Activity against blood sucking mosquitoes<br>- Effect on the microbiome
**Main exclusion/inclusion criteria**	**Inclusion criteria:**<br>- Male or female, aged ≥ 18 years and body weight ≥ 45 kg<br>- *P. falciparum* parasitaemia of 200 to 5000 parasites/µL<br>- Asymptomatic malaria defined as: presence of *P. falciparum* mono-infection with absence of fever (axillary temperature <38.5 °C and absence of history of fever in the recent 24 hours and the week before inclusion) and other symptoms related to malaria<br>- Willingness to take part in the study and to sign the informed consent form<br><br>**Exclusion criteria:**<br>- Active tuberculosis, or history of taking anti-tuberculosis medications within 12 months prior to screening<br>- any *Loa loa microfilaria* infection detected by microscopy<br>- AST/ALT > 2x the upper limit of normal range (ULN)<br>- Taking an experimental drug in the last 4 weeks<br>- Antimalarial treatment in the last 4 weeks<br>- Use of systemic antibiotics with known antimalarial activity within 30 days of study enrolment (e.g. trimethoprim-sulfamethoxazole, doxycycline, tetracycline, clindamycin, erythromycin, fluoroquinolones, or azithromycin).<br>- Use of ivermectin within 30 days of study enrolment<br>- Participants taking herbal medication within one week of screening<br>- Known or suspected electrolyte imbalance, e.g. hypokalaemia, hypocalcaemia or hypomagnesemia with clinical significance<br>- Moderate to severe anaemia (Haemoglobin level <8 g/dL)

	- Any known or suspected immunosuppressive or immunodeficient condition, including human immunodeficiency virus (HIV) infection<br>- Severe malnutrition (Body Mass Index (BMI) < 16.0)<br>- Pregnant or nursing (lactating) women<br>- Known chronic underlying disease such as sickle cell disease or severe cardiac impairment<br>- Participants with serum creatinine ≥ 2 X ULN in the absence of dehydration. In case of dehydration, Participants with serum creatinine ≥ 2 X ULN after oral or parenteral rehydration<br>- Participants with any psychiatric or neurological condition including substance abuse<br>- Allergy to ivermectin
**Study design**	Single-centre, open label and double-blinded, randomized, placebo-controlled dose escalation trial with two stages
**Study population**	Asymptomatic Gabonese adults with confirmed *P. falciparum* infection between 200 and 5000 parasites/µl
**Participant numbers/study treatment arms**	The objective of this study is to assess the safety and activity of escalating doses, most notably a 3-day treatment with 300 µg/kg/day, of ivermectin against asexual parasites of *P. falciparum* versus placebo. Dose escalation is starting with a 200 µg/kg single dose up to a three-day course of 300 µg/kg daily.<br>The first three dose regimens will be tested in five participants each for safety reasons. With this approach there is a 95% probability to detect a relevant Adverse Event occurring at 50% prevalence. If these doses are shown to be safe, for the final stage there will be 17 participants each for the 3x300 µg/kg regimen and for the placebo-control group with appropriate random allocation.<br>To calculate the sample size, we considered the time to 90% parasite reduction of the participants in the 3-day treatment group versus the time to 90% parasite reduction in the placebo group. Based on previous data, we assume that 25% of volunteers allocated to placebo will reduce parasitaemia to 90% of the initial value within 7 days due to natural acquired immunity and expect that at least 75% of ivermectin-treated volunteers will reduce parasitaemia by 90%. To reach a power of 90%, a single-sided alpha of 2.5% and a ratio 1:1 (treatment versus placebo group), 17 participants per group are required.<br><br>Therefore, the following number of participants will be recruited to

	the different treatment arms:<br>In total, 49 participants will be recruited in the study. Arms I-III will be conducted sequentially and only Arm VI will be conducted as a randomized controlled assessment.<br>**<u>Dose escalation procedure:</u>**<br>- Arm I: 5 participants (200 µg/kg single dose)<br>- Arm II: 5 participants (2x200 µg/kg)<br>- Arm III: 5 participants (3x200 µg/kg)<br>Each dose escalation stage will only commence after safety assessment of the previous stage and approval by the Data and Safety Monitoring Board.<br>**<u>Randomized controlled trial:</u>**<br>- Arm IV:<br>- a) 17 participants (3x300 µg/kg)<br>- b) 17 participants (placebo-control)
**Route of Administration**	Oral
**Dose level**	- 200 µg/kg for 1 day<br>- 200 µg/kg for 2 days<br>- 200 µg/kg for 3 days<br>- 300 µg/kg for 3 days
**Treatment duration**	- One day ivermectin<br>- Two days ivermectin<br>- Three days ivermectin<br>- Three days placebo
**Follow-up duration**	14 days (D1, D2, D3, D4, D5, D6, D7, D14)
**Planned Trial Period**	April – December 2019
**Endpoints**	Primary efficacy endpoint<br>1. Time to 90% parasite reduction for at least 8 hours assessed by microscopy<br><br>Secondary efficacy endpoints<br>1. Time to 90% parasite reduction assessed by qPCR<br>2. Difference in AUC of parasitaemia until D7<br>3. Parasite clearance time, defined as time to parasitaemia <100 parasites/mL<br><br>Primary safety endpoint<br>1. Number and occurrence of related SAE and Grade 3 AE from time of first administration of ivermectin until the end of the study

	Secondary safety endpoint<br>1. Number and occurrence of any AE from time of first administration of ivermectin until the end of the study
**Data and Safety Monitoring Plan**	Participants will be treated when criteria to initiate a rescue treatment are reached or at the end of the active study follow-up period (D7).

**Treatment day**		**0**			**1**			**2**			**3**	**4**	**5**	**6**	**7**	**14**
**Time (hours) after first dose**	**Screening**	**0**	**8**	**16**	**24**	**32**	**40**	**48**	**56**	**64**	**72**	**96**	**120**			
Informed consent	X															
Inclusion/ exclusion criteria	X															
Demography, medi-cal history	X															
Haematology and biochemistry	X										X		X		X	X
Asexual & gameto-cyte parasite count (thick & thin blood films)	X	X	X	X	X	X	X	X	X	X	X	X	X	X	X	X
Blood in RNAlater (for qPCR)	X	X	X	X	X	X	X	X	X	X	X	X	X	X	X	X
Blood for membrane feeding	X														X	X
Stool sample	X										X				X	
Vital signs + temper-ature	X	X	X	X	X	X	X	X	X	X	X	X	X	X	X	X
Physical exam	X	X			X			X			X	X	X	X	X	X
Dosing		X			X			X								
PK		X	X	X	X	X	X	X	X	X	X	X	X	X	X	X
Prior and concomi-tant medication	X	X	X	X	X	X	X	X	X	X	X	X	X	X	X	X
AEs	X	X	X	X	X	X	X	X	X	X	X	X	X	X	X	X

Table 3: SCHEDULE OF ASSESSMENTS: Screening to Day 14.
