# Supplementary material for: Efficacy and safety of ivermectin for the treatment of Plasmodium falciparum infections in asymptomatic male and female Gabonese adults – a pilot randomized, double-blind, placebo-controlled single-centre phase Ib/IIa clinical trial
Source: eBioMedicine. 2023 Oct 13;97:104814. doi: 10.1016/j.ebiom.2023.104814 (PMC10582777; doi:10.1016/j.ebiom.2023.104814)
Supplement: DSMB charter [file mmc11.pdf]

cermel
Centre de Recherches Médicales de Lambaréné

www.cermel.org
BP 242 · Lambaréné
BP 1437 · Libreville
Gabon
Tel: +241 07 98 91 91
admin@cermel.org

IVERCURE

Data and Safety Monitoring Board Charter

**Clinical Study/Trial title:**	*Efficacay and safety of Ivermectin for the treatment of Plasmodium falciparum infection in asymptomatic Gabonese adults.*
**Study protocol version this charter is based on:**	Protocol version 3 (30 April 2019)
**Principal Investigator: & Contact information:**	Dr. Rella Zoleko Manego<br>Department of Clinical Operation<br>CERMEL<br>Lambaréné
**Ethics registration num-ber:**	CEI/CERMEL 006/2019
**Trial registration number:**	PACTR201908520097051

**DSMB Charter Version No.**	V3
**Effective Date of DSMB Charter:**	06 April 2020

Participants will be included sequentially in treatment arms I-IV with a pause between arms to allow for safety review by the DSMB. Each dose escalation stage will only commence after safety assessment of the previous study dose regimen and approval by the DSMB. A final DSMB interim safety analysis will be performed before start of the randomized trial stage.

Safety of ivermectin treatment will be assessed by analysing the frequency, incidence and nature of adverse events and serious adverse events arising during the study. As per trial protocol, the safety endpoints to be evaluated are:

- Primary: Number and occurrence of at least possibly related SAE and Grade 3 AE from time of first administration of ivermectin until D7.
- Secondary: Number and occurrence of any AE from time of first administration of ivermectin until D7.

www.cermel.org
BP 242 · Lambaréné
BP 1437 · Libreville
Gabon
Tel: +241 07 98 91 91
admin@cermel.org

The sponsor can, in consultation with the DSMB, suspend recruitment at any time based on safety considerations. The DSMB is to counsel the sponsor on this. Furthermore, the DSMB can recommend measures to improve assessment of safety outcomes, such as additional clinical tests, and can propose protocol amendments, if needed.

In order to avoid bias, no interim safety assessment shall be performed during the randomized trial stage if there are no possibly or probably related SAE or grade 3 AE.
