# Supplementary material for: Efficacy and safety of ivermectin for the treatment of Plasmodium falciparum infections in asymptomatic male and female Gabonese adults – a pilot randomized, double-blind, placebo-controlled single-centre phase Ib/IIa clinical trial
Source: eBioMedicine. 2023 Oct 13;97:104814. doi: 10.1016/j.ebiom.2023.104814 (PMC10582777; doi:10.1016/j.ebiom.2023.104814)
Supplement: Protocol [file mmc12.pdf]

Clinical Trial Protocol

Efficacy and safety of Ivermectin for the treatment of *Plasmodium falciparum* infections in asymptomatic Gabonese adults

Protocol version 4 (24-06-2019)

(with Amendment: additional blood sampling for membrane feeding on hours 52 and 72)

Trial Sponsor:	Centre de Recherches Médicales de Lambaréné (CERMEL)
Investigator:	Rella Zoleko-Manego, MD, MSc
Coordinating Investigator:	Jana Held, PhD
Co-Investigator:	Ghyslain Mombo-Ngoma, MD, PhD
Authors:	Lais Carvalho
	Dorothea Sträßner
	Wilfrid Ndoumba, MD
	Lia Betty Dimessa, MD
	Malick Akinosho, MD
	Mirjam Groger, MD, PhD
	Sebastian Wicha
Statistician:	Benjamin Mordmüller, MD, PhD

Safety Monitor: Michael Ramharter, MD, MSc, DTMH

Clinical Trial Sponsor:

Centre de Recherches Médicales de Lambaréné (CERMEL)

B.P. 242, Lambaréné

Gabon

Represented by:

Ayôla Akim Adegnika

Modification history

**Version**	**Date**	**Major Changes**	**Authors**
Version 4	24 June 2019	Blood sample for membrane feeding on hours 52 and 72	Jana Held, Dorothea Sträßner, Rella Zoleko-Manego, Benjamin Mordmüller, Michael Ramharter, Ghyslain Mombo-Ngoma
Version 3	30 April 2019	follow-up day 14 added	Dorothea Sträßner, Rella Zoleko-Manego, Jana Held, Benjamin Mordmüller, Michael Ramharter, Ghyslain Mombo-Ngoma
Version 2	08 April 2019	dosing scheme adjusted	Dorothea Sträßner, Rella Zoleko-Manego, Jana Held, Benjamin Mordmüller,

			Michael Ramharter, Ghyslain Mombo-Ngoma
Version 1	22 February 2019		Dorothea Sträßner, Lais Carvalho, Rella Zoleko-Manego, Jana Held, Benjamin Mordmüller, Michael Ramharter, Ghyslain Mombo-Ngoma

Table of contents

1. Synopsis 6
2. Abbreviations 11
3. Background and Rationale 12
   3.1. Epidemiology and consequences of malaria 12
   3.2. Endectocides and ivermectin 12
   3.3. Safety profile of ivermectin 12
   3.4. Ivermectin as additional tool in malaria control 13
4. Trial design 14
   4.1. Trial overview 14
   4.2. Objectives 15
      4.2.1. Primary objective 15
      4.2.2. Secondary objectives 15
      4.2.3. Exploratory objectives 16
5. Study population 16
   5.1. Sample size 16
   5.2. Inclusion criteria 16
   5.3. Exclusion criteria 17

5.4. Recruitment and Informed Consent ... 17
5.5. Withdrawal of Participants ... 18
6. Study procedures ... 19
6.1. Screening visit ... 19
6.2. Treatment ... 19
6.2.1. Treatment arms ... 19
6.2.2. Randomization of treatment ... 20
6.2.3. Blinding and treatment procedure ... 20
6.2.4. Concomitants medication ... 21
6.2.5. Prohibited medication ... 21
6.2.6. Rescue medication ... 21
7. Routine assessment ... 22
7.1. Efficacy assessment ... 24
7.2. Safety Assessment ... 24
7.3. Exploratory Assessments ... 24
8. Endpoints ... 24
8.1. Primary Efficacy Endpoint ... 24
8.2. Secondary Efficacy Endpoints ... 24
8.3. Primary Safety Endpoint ... 25
8.4. Secondary Safety Endpoint ... 25
9. Duration of the study ... 25
10. Potential risks for participants ... 25
10.1. Phlebotomy ... 25
10.2. *Plasmodium falciparum* infection ... 25
10.3. Treatment with ivermectin: ... 26
11. Potential benefits for participants ... 26
12. Safety assessment ... 27
12.1. Definitions ... 27

12.2. Causality assessment 28
12.3. Reporting procedures for AEs (Excluding SAEs) 29
12.4. Reporting procedures for SAEs 29
12.5. Reporting Procedures for SUSARS 30
12.6. Safety hold of the study and stopping rules 30
13. Quality control and quality assurance procedures 30
14. Ethics 30
14.1. Informed Consent 30
14.2. Ethics review 31
14.3. Participant Confidentiality 31
15. Data handling and record keeping 31
15.1. Data Handling 31
15.2. Record Keeping 31
15.3. Source Data and Case Report Forms (CRFs) 31
15.4. Data Protection 32
15.5. Procedures for the collection, storage of biological sample 32
16. References 33

1. Synopsis

**Study title**	Efficacy and safety of Ivermectin for the treatment of *Plasmodium falciparum* infections in asymptomatic Gabonese adults
**Study acronym**	**IVERCURE**
**Protocol version**	V.4
**Protocol date**	24 June 2019
**Clinical Phase**	1
**Trial Centre(s)**	CERMEL, Gabon
**Rationale**	Ivermectin has a potent anti-parasitic and anti-insecticide activity against many organisms including ecto- and endoparasites in animals and in humans.<br><br>Malaria, a mosquito-borne disease caused by parasites of the genus *Plasmodium*, remains the most important parasitic disease in humans worldwide. It has been shown that ivermectin can reduce transmission of *Plasmodium* parasites by its activity against blood-sucking mosquitoes, but recent *in vitro* data suggests that ivermectin has also and effect on the erythrocytic stages of *P. falciparum*, <u>but *in vivo* data are lacking.</u><br><br>Besides its insecticidal activity, the excellent safety profile of ivermectin, which has been used for decades in mass drug administration programs, and a different mechanism of action compared to other antimalarials make ivermectin an interesting candidate for malaria control and elimination campaigns.<br><br>This dose escalation study is designed to assess the activity of ivermectin on the parasitaemia of asymptomatic *P. falciparum* infections. The aim of this study is to investigate whether ivermectin is safe at a 3x300 µg/kg treatment course and can reduce or clear parasitaemia in participants infected with *P. falciparum* assessed by thick blood smear microscopy.
**Primary Objective(s)**	- To assess the safety and tolerability of single- and multiple ascending doses of ivermectin in volunteers with asymptomatic *P. falciparum* infection<br>- To assess the efficacy of ivermectin in participants with asymptomatic *P. falciparum* infection

**Secondary Objective(s)**	- To compare efficacy of ivermectin 200µg/kg single dose versus two-day 200µg/kg treatment versus three-day 200µg/kg treatment versus three-day 300µg/kg treatment<br>- To compare efficacy of ivermectin three-day 300µg/kg treatment compared to placebo<br>- To compare safety and tolerability of ivermectin 200µg/kg single dose versus two-day 200µg/kg treatment versus three-day 200µg/kg treatment versus three-day 300µg/kg treatment<br>- To compare safety and tolerability of ivermectin three-day 300µg/kg treatment compared to placebo
**Exploratory Objectives**	To assess:<br>- Effect of pharmacokinetic parameters on efficacy on safety<br>- Activity of ivermectin on *P. falciparum* gametocytes<br>- Parasite kinetics in the placebo group<br>- Activity against blood sucking mosquitoes<br>- Effect on the microbiome
**Main exclusion/inclusion criteria**	**Inclusion criteria:**<br>- Male or female, aged ≥ 18 years and body weight ≥ 45 kg<br>- *P. falciparum* parasitaemia of 200 to 5000 parasites/µL<br>- Asymptomatic malaria defined as: presence of *P. falciparum* mono-infection with absence of fever (axillary temperature <38.5 °C and absence of history of fever in the recent 24 hours and the week before inclusion) and other symptoms related to malaria<br>- Willingness to take part in the study and to sign the informed consent form<br><br>**Exclusion criteria:**<br>- Active tuberculosis, or history of taking anti-tuberculosis medications within 12 months prior to screening<br>- any *Loa loa microfilaria* infection detected by microscopy<br>- AST/ALT > 2x the upper limit of normal range (ULN)<br>- Taking an experimental drug in the last 4 weeks<br>- Antimalarial treatment in the last 4 weeks<br>- Use of systemic antibiotics with known antimalarial activity within 30 days of study enrolment (e.g. trimethoprim-sulfamethoxazole, doxycycline, tetracycline, clindamycin, erythromycin, fluoroquinolones, or azithromycin).<br>- Use of ivermectin within 30 days of study enrolment<br>- Participants taking herbal medication within one week of screening

	- Known or suspected electrolyte imbalance, e.g. hypokalaemia, hypocalcaemia or hypomagnesemia with clinical significance<br>- Moderate to severe anaemia (Haemoglobin level <8 g/dL)<br>- Any known or suspected immunosuppressive or immunodeficient condition, including human immunodeficiency virus (HIV) infection<br>- Severe malnutrition (Body Mass Index (BMI) < 16.0)<br>- Pregnant or nursing (lactating) women<br>- Known chronic underlying disease such as sickle cell disease or severe cardiac impairment<br>- Participants with serum creatinine ≥ 2 X ULN in the absence of dehydration. In case of dehydration, Participants with serum creatinine ≥ 2 X ULN after oral or parenteral rehydration<br>- Participants with any psychiatric or neurological condition including substance abuse<br>- Allergy to ivermectin
**Study design**	Single-centre, open label and double-blinded, randomized, placebo-controlled dose escalation trial with two stages
**Study population**	Asymptomatic Gabonese adults with confirmed *P. falciparum* infection between 200 and 5000 parasites/µl
**Participant numbers/study treatment arms**	The objective of this study is to assess the safety and activity of escalating doses, most notably a 3-day treatment with 300 µg/kg/day, of ivermectin against asexual parasites of *P. falciparum* versus placebo. Dose escalation is starting with a 200 µg/kg single dose up to a three-day course of 300 µg/kg daily.<br>The first three dose regimens will be tested in five participants each for safety reasons. With this approach there is a 95% probability to detect a relevant Adverse Event occurring at 50% prevalence. If these doses are shown to be safe, for the final stage there will be 17 participants each for the 3x300 µg/kg regimen and for the placebo-control group with appropriate random allocation.<br>To calculate the sample size, we considered the time to 90% parasite reduction of the participants in the 3-day treatment group versus the time to 90% parasite reduction in the placebo group. Based on previous data, we assume that 25% of volunteers allocated to placebo will reduce parasitaemia to 90% of the initial value within 7 days due to natural acquired immunity and expect that at least 75% of ivermectin-treated volunteers will reduce parasitaemia by 90%. To reach a power of 90%, a single-sided alpha of 2.5% and a ratio 1:1 (treatment versus placebo group), 17 participants per group are required.

	Therefore, the following number of participants will be recruited to the different treatment arms:<br>In total, 49 participants will be recruited in the study. Arms I-III will be conducted sequentially and only Arm VI will be conducted as a randomized controlled assessment.<br>**<u>Dose escalation procedure:</u>**<br>- Arm I: 5 participants (200 µg/kg single dose)<br>- Arm II: 5 participants (2x200 µg/kg)<br>- Arm III: 5 participants (3x200 µg/kg)<br>Each dose escalation stage will only commence after safety assessment of the previous stage and approval by the Data and Safety Monitoring Board.<br>**<u>Randomized controlled trial:</u>**<br>- Arm IV:<br>- a) 17 participants (3x300 µg/kg)<br>- b) 17 participants (placebo-control)
**Route of Administration**	Oral
**Dose level**	- 200 µg/kg for 1 day<br>- 200 µg/kg for 2 days<br>- 200 µg/kg for 3 days<br>- 300 µg/kg for 3 days
**Treatment duration**	- One day ivermectin<br>- Two days ivermectin<br>- Three days ivermectin<br>- Three days placebo
**Follow-up duration**	14 days (D1, D2, D3, D4, D5, D6, D7, D14)
**Planned Trial Period**	April – December 2019
**Endpoints**	Primary efficacy endpoint<br>1. Time to 90% parasite reduction for at least 8 hours assessed by microscopy<br><br>Secondary efficacy endpoints<br>1. Time to 90% parasite reduction assessed by qPCR<br>2. Difference in AUC of parasitaemia until D7<br>3. Parasite clearance time, defined as time to parasitaemia <100 parasites/mL

	Primary safety endpoint<br>1. Number and occurrence of related SAE and Grade 3 AE from time of first administration of ivermectin until the end of the study<br><br>Secondary safety endpoint<br>1. Number and occurrence of any AE from time of first administration of ivermectin until the end of the study
**Data and Safety Monitoring Plan**	Participants will be treated when criteria to initiate a rescue treatment are reached or at the end of the active study follow-up period (D7).

2. **Abbreviations**

AE	Adverse Event
ALT	Alanine aminotransferase
AST	Aspartate transaminase
CBC	Complete Blood Count
CRP	C Reactive Protein
DSMB	Data and Safety Monitoring Board
EC	Ethics Committee
GCP	Good Clinical Practice
GGT	Gamma-Glutamyl Transpeptidase
HIV	Human Immunodeficiency Virus
ICH	International Conference on Harmonisation

3. Background and Rationale

3.1. Epidemiology and consequences of malaria

In spite of remarkable progress over the last 15 years (1), malaria continues to be a major public health problem in the developing world with an estimated 219 million cases and 435.000 deaths in 2017 (2), an incidence that has levelled off over the last years. The enormous economic and social consequences of malaria have been well documented (3). By far the major burden is in Africa and the enormous economic and social consequences of malaria have been well documented (3).

3.2. Endectocides and ivermectin

Ivermectin is an anti-parasitic medicine approved for the treatment and control of human onchocerciasis, lymphatic filariasis, strongyloidiasis and scabies (4). It is also an endectocide, a systemic insecticide capable of killing mosquitoes that feed on treated participants regardless of the time and place of biting (5). Ivermectin mass drug administration could circumvent residual transmission and potentially become a complementary tool for malaria elimination. One key issue is the classification of this potential endectocide-based intervention. Many policy-makers consider it as an altruistic intervention in which the main benefit from exposing an individual to the drug is experienced at community level once mosquitoes die. Other authors consider a reduction in local transmission as a personal benefit that warrants mass treatment. In the case of ivermectin, additional personal benefits are expected from its effects on helminths and ectoparasites (6). Within the current study the activity of ivermectin against the blood stage parasite of *P. falciparum* will be investigated, this would present an additional benefit for the individual person in case ivermectin will be widely used for malaria transmission blocking. In addition, ivermectin shall have a different mechanism of action than other antimalarials and therefore constitute a particularly interesting combination partner for malaria treatments, especially for use in malaria control and elimination settings as well as programs targeting co-infections.

3.3. Safety profile of ivermectin

Ivermectin has been licensed for human use for almost 30 years, and its safety has been assessed in over 70 trials. More than 2.7 billion 150-200 mcg/kg single doses have been distributed through the Mectizan Donation program (7). In individuals with a high *Loa loa* microfilarial load, Ivermectin can cause severe encephalopathy (6). Outside *Loa loa* endemic areas, the drug is remarkably safe. In onchocerciasis-infected patients, adverse events (AE) to ivermectin are

usually mild, transient, associated with intensity of microfilarial infection and primarily characterized as mild Mazzoti-type reactions to dying microfilaria (8). These effects wane in subsequent administrations (9). No significant dose-response has been found between ivermectin plasma levels and AEs in human dose-ranging studies (10). A recent Cochrane review of ivermectin for river blindness shows that side effects are rarely reported (11). The French authorities recommend up to 400 µg/kg for the control of lymphatic filariasis (12). Some studies showed that also higher doses of ivermectin were well tolerated in adults with multiple doses up to 3 days of 600 µg/kg per day or up to a single dose of 2000 µg/kg (13–16). Currently there is evidence for a three-day course of 300 µg/kg ivermectin to be the most promising regimen for future mass drug administrations (14).

For severe crusted scabies, up to seven 200 µg/kg doses within a month in combination with topical treatment and keratolytics are recommended in the US (17) and Australia (18). The possibility of using more than 3 doses for the treatment of moderate to severe crusted scabies cases is included in the Australian product information (IPC-MK0933-T-062010) (4). Pharmacokinetic investigations of ivermectin single dose (12 mg) showed a Tmax at 4.4 hours post-dose and a half-life of 38.9 hours (31).

3.4. Ivermectin as additional tool in malaria control

Recently, ivermectin has emerged as a potential new additional tool for malaria transmission blocking and control (19,20) because of its capacity to inhibit parasite transmission (21–23) and sporogony (24–26), and its insecticidal activity (27–30). Personal data (publication pending) suggest that ivermectin is also active against asexual stages (Table 1) and mature gametocytes (Table 2) *in vitro.*

**Drug-sensitivity assay**		
**Parasite strains**	**Drugs (IC50 ± SD in nM)**	
	Chloroquine	Ivermectin
3D7	3.3 ± 1.5	100 ± 32.1
Dd2	206.9 ± 52.7	110 ± 42.2
JH1	103 ±20.6	21.5 ±6.9
JH13	111.2 ± 21	126.4 ± 40.6

JH26	10.9 ± 4.2	137.9 ± 38.2
K1	321.7 ± 72.1	365.3 ± 92.3

Table 1: Results of the asexual growth inhibition assay for chloroquine and ivermectin presented as IC50 in nM. The assay was performed at least three times in duplicate. The mean ± standard deviation is given.

**Drug-sensitivity assay**	
**Drugs**	**IC50 ± SD (nM)**
Chlorotonil A	6.43 ± 3.6
Methylene Blue	284.9 ± 203.4
Epoxomicin	2.7 ± 1.7
Ivermectin	558.7 ± 103.9

Table 2: IC 50 values in nM of different compounds against mature gametocytes of *P. falciparum*. The assay was performed in duplicate for at least three times. The mean ± the standard deviation is given.

A clinical trial in asymptomatic infected adult participants will provide valuable data regarding this potential therapeutic effect. As reference, the Cmax reached after a single dose of 400 µg/kg accompanied by a standard fatty diet is 260 ng/ml (16), this is approximately 3x the 50% inhibitory concentration for most strains. The JH strains (JH1, JH13, JH26) are clinical isolates collected in 2009 from Participants from Lambaréné, Gabon adapted to continuous culture. An IC50 value of 100 nM corresponds to 87 ng/mL, a concentration that can be kept for 12 h when an oral single dose of 30 mg of ivermectin is given with food, therefore the *in vitro* inhibitory effects could also be translated to an *in vivo* effect. One of the potential molecular explanations for the activity of ivermectin in *P. falciparum* is the inhibition of the nuclear import of signal recognition particle components (30). The results of this phase 1 proof-of-concept trial should back decisions about future ivermectin trials in Gabon.

4. Trial design

4.1. Trial overview

The study is designed as a dose escalation trial with who stapes to assess the safety and efficacy of ivermectin on the reduction of *P. falciparum* parasitaemia in asymptomatic participants in

an endemic area of Gabon. The first stage is an open label trial and included arms I-III. The next stage is a randomized double-blind controlled trial. Potential adult participants living in Lambaréné and in the Tsamba-Magotsi Department, Gabon will be screened for parasitaemia of 200 to 5000 *P. falciparum* parasites/µl without clinical signs of malaria for inclusion in the clinical trial. Participants will be included sequentially in arms I-IV with a pause between arms to allow safety review by DSMB. Each dose escalation stage will only commence after safety assessment of the previous stage and approval by the Data and Safety Monitoring Board. The sponsor can, in consultation with the DSMB, suspend recruitment at any time based on safety considerations.

Study arms:

- Arm I: 5 participants (200 µg/kg single dose)
- Arm II: 5 participants (2x200 µg/kg)
- Arm III: 5 participants (3x200 µg/kg)
- Arm IV:
    a) 17 participants (3x300 µg/kg)
    b) 17 participants (placebo-control)

All participants will be hospitalized for three days. Assessment during treatment period and follow-up visit will be the same in all study arms.

4.2. Objectives

4.2.1. Primary objective

- To assess the safety and tolerability of single- and multiple ascending doses of ivermectin in volunteers with asymptomatic *P. falciparum* infection
- To assess the efficacy of ivermectin in participants with asymptomatic *P. falciparum* infection

4.2.2. Secondary objectives

- To compare efficacy of ivermectin 200µg/kg single dose versus two-day 200µg/kg treatment versus three-day 200µg/kg treatment versus three-day 300µg/kg treatment
- To compare efficacy of ivermectin three-day 300µg/kg treatment compared to placebo
- To compare safety and tolerability of ivermectin 200µg/kg single dose versus two-day 200µg/kg treatment versus three-day 200µg/kg treatment versus three-day 300µg/kg treatment
- To compare safety and tolerability of ivermectin three-day 300µg/kg treatment compared to placebo

4.2.3. Exploratory objectives

- To assess effect of pharmacokinetic parameters on efficacy and safety
- To assess activity of ivermectin on *P. falciparum* gametocytes
- To assess parasite kinetics in the placebo group
- To assess the mosquitocidal effect
- To assess the effect on the metabolome. As ivermectin has an influence on the worm burden stool sample analysis will enable to relate this to metabolomic changes.

5. Study population

5.1. Sample size

Treatment Arms I-III serves to cautiously assess the safety of Ivermectin against *Plasmodium falciparum* infections and the sample size for these treatment arms is purely descriptive.

As in Arm IV the currently envisaged dose for ivermectin mass drug administrations in malaria elimination programs will be assessed, this arm has a formal sample size and placebo-controls. To calculate the sample size, we considered the time to 90% parasite reduction of the participants in the 3-day treatment group versus the time to 90% parasite reduction in the placebo group. Based on previous data, we assume that 25% of volunteers allocated to placebo will reduce parasitaemia to 90% of the initial value within 7 days and expect that at least 75% of ivermectin-treated volunteers will reach the endpoint. With a power of 90%, a single-sided alpha of 2.5% and a ratio 1:1 (treatment versus placebo group), 17 events (achieving 90% parasite reduction) are required (32). Consequently, 17 volunteers will be recruited per arm. Therefore, a total of 49 evaluable participants is required. Participants who are lost to follow up will be included in the safety analysis, but replacements will be recruited to reach the number of 49 evaluable participants.

5.2. Inclusion criteria

Participants eligible for inclusion in this study must fulfil all of the following criteria:

- Male or female, aged ≥ 18 years and body weight ≥ 45 kg
- *P. falciparum* parasitaemia of 200 to 5000 parasites/µL
- Asymptomatic *P. falciparum* infection defined as: presence of *P. falciparum* mono-infection with absence of fever (axillary temperature <38.5 °C and absence of history of fever in recent 24 hours and the week before inclusion) and other symptoms related to malaria.

- Willingness to take part in the study and to sign the informed consent form

5.3. Exclusion criteria

Participants fulfilling any of the following criteria are not eligible for inclusion in this study

- Active tuberculosis, or history of taking anti-tuberculosis medications within 12 months prior to screening
- any *Loa loa microfilaria* infection detected by microscopy
- AST/ALT > 2x the upper limit of normal range (ULN)
- Taking an experimental drug in the last 4 weeks
- Antimalarial treatment in the last 4 weeks
- Use of systemic antibiotics with known antimalarial activity within 30 days of study enrolment (e.g. trimethoprim-sulfamethoxazole, doxycycline, tetracycline, clindamycin, erythromycin, fluoroquinolones, or azithromycin).
- Use of ivermectin within 30 days of study enrolment
- Participants taking herbal medication within one week of screening
- Known or suspected electrolyte imbalance, e.g. hypokalaemia, hypocalcaemia or hypomagnesemia with clinical significance
- Moderate to severe anaemia (Haemoglobin level <8 g/dL)
- Any confirmed or suspected immunosuppressive or immunodeficient condition, including human immunodeficiency virus (HIV) infection
- Severe malnutrition (Body Mass Index (BMI) < 16.0)
- Pregnant or nursing (lactating) women
- Known chronic underlying disease such as sickle cell disease or severe cardiac impairment
- Participants with serum creatinine ≥ 2 X ULN in the absence of dehydration. In case of dehydration, Participants with serum creatinine ≥ 2 X ULN after oral or parenteral rehydration
- Participants with any psychiatric or neurological condition including substance abuse
- Allergy to ivermectin

5.4. Recruitment and Informed Consent

All participants will sign and date the informed consent form before any study specific procedure is performed. At the screening visit, the participant will be fully informed of all aspects of

the trial, the potential risks and their obligations. The following general principles will be emphasized:

- Participation in the study is entirely voluntary
- Refusal to participate involves no penalty or loss of medical benefits
- The participant may withdraw from the study at any time
- The participant is free to ask questions at any time to allow him or her to understand the purpose of the study and the procedures involved

The aims of the study and all tests to be carried out will be explained. The participant will be given the opportunity to ask about the details of the trial and will then have sufficient time to consider whether or not to participate. Participants will be asked to sign and date two copies of the consent form, one which will belong to them to keep, and one to be stored in the Investigator`s File. These forms will also be signed and dated by the Investigator.

5.5. Withdrawal of Participants

In accordance with the principles of the current revision of the Declaration of Helsinki (updated 2013) and any other applicable regulations, a participant has the right to withdraw from the study at any time and for any reason and is not obliged to give his or her reasons for doing so. The Investigator may withdraw the participant at any time in the interests of the participant`s health and well-being. In addition, the participant may withdraw/be withdrawn for any of the following reasons:

- Investigator decision
- An AE, which requires discontinuation of the study involvement or results in inability to continue to comply with study procedures

The reason for withdrawal will be recorded in the CRF. If withdrawal is due to an AE, appropriate follow-up visits or medical care will be arranged, with the agreement of the participant, until the AE has resolved, stabilized or a non-trial related causality has been assigned. Any participant who is withdrawn from the study may be replaced, if that is possible within the specified time frame.

If a participant withdraws from the study, blood samples collected before their withdrawal from the trial will be used/ stored unless the participant specifically requests otherwise. The samples may be destroyed if the participant did not agree to have their samples stored.

6. Study procedures

6.1. Screening visit

Once the informed consent is signed, the following screening procedures will be undertaken:

- Full medical history

- Prior concomitant medication

- Demographic data (gender, age)

- Clinical examination (including measurement of height and weight)

- Vital signs (including pulse, blood pressure and body temperature)

- Pregnancy test in female participants of childbearing potential (18 – 49 years)

- Biochemistry tests: AST, ALT and creatinine,

- Haematology: full blood count and WBC differential blood count

- Malaria blood film for parasite count and species as well as identification of *Loa loa*

- Blood sampling for qPCR

AEs will be recorded from the signature of the informed consent. Abnormal clinical findings from the medical history, vital signs assessment or blood tests at any point in the study will be assessed using established reference intervals of the laboratory. If an abnormal finding is deemed to be clinically significant, the participant will be informed, and appropriate medical care arranged with the permission of the participant.

6.2. Treatment

6.2.1. Treatment arms

Participants fulfilling all inclusion criteria and none of the exclusion criteria will be assigned sequentially to receive ivermectin 200 µg/kg orally single dose for one (5 first subjects), two or three days (10 next available subjects). Following interim safety analysis, 34 subjects will be randomly allocated in two treatment arms to receive either 300 µg/kg per day for 3 days or placebo for 3 days. There will be four consecutive study arms (arms IV a) and b) will be parallel):

- Arm I: 5 participants (200 µg/kg single dose)
- Arm II: 5 participants (2x200 µg/kg)

- Arm III: 5 participants (3x200 µg/kg)
- Arm IV:
    a) 17 participants (3x300 µg/kg)
    b) 17 participants (placebo-control)

6.2.2. Randomization of treatment

As Arms I-III serves descriptive purposes for safety assessment of the study drug, there will be no randomization.

Participants for Arm IV will be randomized to arm IVa or arm IV b through a randomization system prior to the first treatment. The randomization ratio will be 1:1 (3-day treatment, control group). A dedicated member of the team, who is not involved in volunteer management or diagnostic activities, will keep the randomization envelopes and dosing schedule. A third party outside the study team and sponsor will generate and distribute the randomization list and sealed envelopes using a random number generator.

A sealed envelope containing the participants arm will be used for randomization in the study and the randomization data are kept strictly confidential until formal interim safety review or the final analysis. At the time of final analysis, the allocation data will be loaded into the statistical programming system for access to trial statisticians and pharmacokinetics.

6.2.3. Blinding and treatment procedure

One none-blinded person will prepare the treatment assigned by the randomization system, verify that the Participant's treatment group is assigned by the envelope and complete the corresponding treatment record form. Only the person administering ivermectin and placebo would know the groups allocation. Ivermectin and placebo are similar but not identical and will be administered to each participant separately out of the original packaging without changing the labels. All other study personnel (e.g. physicians, microscopists) and study participants will be unaware of the group allocation.

The study drugs will be administered under supervision with a fat containing food such as a croissant with bread spread. These two persons (the person administering the drug and the supervising person) are not otherwise involved in the conduct of the trial. The first dose will be administered at the time of the participant’s recruitment into the study. Time between the blood film preparation and treatment administration will be no longer than four hours. Subsequent doses will be given at twenty-four hourly intervals, food will be provided together with the investigational drug.

In the event of vomiting/spitting within one hour of ingestion, the same dose of drug will be re-administered from a supplemental drug supply. However, re-dosing will be done only once per day of treatment administration.

All study treatment taken must be recorded in the CRF, along with any comments about whether the Participants swallowed all or part of the medication.

6.2.4. Concomitants medication

All medication (other than the investigational drugs) and significant non-drug therapies administered after the Participant starts treatment with study drug will be documented on the concomitant medication.

6.2.5. Prohibited medication

The use of the following treatments is NOT allowed

- Antimalarials other than the study drug and rescue medication (Artemether/lumefantrine) are not permitted during the entire study period.
- Herbal medication should not have been taken within 1 week before the study treatment is given and should be avoided until the last study visit

As ivermectin is metabolized by Cytochrome P450 3A4 (abbreviated CYP3A4), especially substances which could alter Cyp3A4 activity should be avoided before and after intake of ivermectin. This includes substrates as well as inhibitors and inducers of Cyp3A4. Only participants willing to avoid the intake of these substances before and after administration of ivermectin will be enrolled in the study.

6.2.6. Rescue medication

The following circumstances warrant discontinuation of study treatment and the implementation of rescue medication

- Any parasitaemia based on microscopy with fever (>38.5°C, observed by study team) post-dose
- Development of danger signs or severe malaria on any treatment day between Study Day 0 and Day 7 in the presence of parasitaemia
- Parasitaemia ≥ 20,000 parasites/µl

The start of the rescue medication with artemether-lumefantrine may occur after the start of trial medications and up to Study Day 7 as deemed necessary by the investigator. Any

participants who would not need the rescue medication up to study Day 7, will be given artemether-lumefantrine at end of active follow-up (Day 7). The rescue medication according to the local guidance will be used to treat severe malaria.

7. Routine assessment

All clinical visits and procedures will be undertaken by one investigator of the clinical team. The procedures to be included in each visit are documented in Table 1. Each procedure is assigned a time point and a window period of ± 4 hours within which the review will be conducted. Participants have to be seen at all visits on the designated day, or as close to it as possible.

**Treatment day**		**0**			**1**			**2**			**3**	**4**	**5**	**6**	**7**	**14**
**Time (hours) after first dose**	**Screen-ing**	**0**	**8**	**16**	**24**	**32**	**40**	**48**	**56**	**64**	**72**	**96**	**120**			
Informed consent	X															
Inclusion/ exclusion criteria	X															
Demography, medi-cal history	X															
Haematology and biochemistry	X										X		X		X	X
Asexual & gameto-cyte parasite count (thick & thin blood films)	X	X	X	X	X	X	X	X	X	X	X	X	X	X	X	X
Blood in RNAlater (for qPCR)	X	X	X	X	X	X	X	X	X	X	X	X	X	X	X	X
Blood for membrane feeding<br>*4 hours after last ivermectin treat-ment together with PK sample	X								X*		X				X	X
Stool sample	X										X				X	
Vital signs + tem-perature	X	X	X	X	X	X	X	X	X	X	X	X	X	X	X	X
Physical exam	X	X			X			X			X	X	X	X	X	X
Dosing		X			X			X								
PK		X	X	X	X	X	X	X	X	X	X	X	X	X	X	X

Prior and concomitant medication	X	X	X	X	X	X	X	X	X	X	X	X	X	X	X	X
AEs	X	X	X	X	X	X	X	X	X	X	X	X	X	X	X	X

Table 3: SCHEDULE OF ASSESSMENTS: Screening to Day 14.

7.1. Efficacy assessment

- Parasitaemia (thick and thin blood smears) on screening, Days 0 (H0, H8, H16), 1 (H24, H32, H40), 2 (H48, H56, H64), 3 (H72), 4, 5, 6, 7 and 14
- qPCR on screening, Days 0 (H0, H8, H16), 1 (H24, H32, H40), 2 (H48, H56, H64), 3 (H72), 4, 5, 6, 7 and 14

7.2. Safety Assessment

- Physical examinations at screening and on H0, H24, H48, H72, and days 4, 5, 6, 7 and 14
- Vital signs, including pulse rate and blood pressure and temperature on screening, Days 0 (H0, H8, H16), 1 (H24, H32, H40), 2 (H48, H56, H64), 3 (H72), 4, 5, 6, 7 and 14
- Haematology, biochemistry analysis at screening, day 3 (H72), day 5, 7 and 14
- PK on Days 0 (H0, H8, H16), 1 (H24, H32, H40), 2 (H48, H56, H64), 3 (H72), 4, 5, 6, 7 and 14
- Recording of adverse events and concomitant medication on all time points

Participants must be seen at all visits on the designated day, or as close to it as possible. Missed or rescheduled visits should not lead to automatic discontinuation.

7.3. Exploratory Assessments

- Stool sample at screening and on days 3 (H72) and 7
- Blood sample for membrane feeding at screening, hour 52 representing Cmax of ivermectin (4 hours after last ivermectin treatment), hour 72 and on days 7 and 14

8. Endpoints

8.1. Primary Efficacy Endpoint

1. Time to 90% parasite reduction for at least 8 hours assessed by microscopy

8.2. Secondary Efficacy Endpoints

1. Time to 90% parasite reduction assessed by qPCR

2. Difference in AUC of parasitaemia until D7
3. Parasite clearance time, defined as time to parasitaemia <100 parasites/mL

8.3. Primary Safety Endpoint

1. Number and occurrence of related SAE and Grade 3 AE from time of first administration of ivermectin until the end of the study

8.4. Secondary Safety Endpoint

1. Number and occurrence of any AE from time of first administration of ivermectin until the end of the study

9. Duration of the study

Participants have to be hospitalized for a at least 72 hours or for up to 7 days, depending on their convenience. After discharge from the hospital, Participants will return daily to the Clinical Centre for further assessments during active follow-up until Day 7 and a final visit on Day 14. The duration of involvement in the study from day 0 (day of ivermectin administration) will be 14 days. The start of the trial is defined as the date of the first visit of the first participant. The end of the trial is the date of the last visit of the last participant.

10. Potential risks for participants

10.1. Phlebotomy

The maximum volume of blood drawn over the study period is approximately 300 ml over 14 days. Additional blood samples could be required for safety reasons. However, this volume should not compromise these participants. There may be minor bruising, local tenderness or presyncopal symptoms associated with venepuncture, which will not be documented as AEs if they occur. Rare possible side effects are infections, thrombophlebitis and nerval lesions.

10.2. *Plasmodium falciparum* infection

Even asymptomatic participants can develop malaria during the clinical trial. Symptoms and signs may include fever, tachycardia, hypotension, chills, rigors, sweats, headache, anorexia, nausea, vomiting, diarrhoea, myalgia, arthralgia, low back pain, thrombocytopenia and lymphopenia. All participants will be closely followed up during the clinical trial and will be treated at the end of study.

10.3. Treatment with ivermectin:

Ivermectin is a drug with category C during pregnancy. In Participants treated for strongyloidiasis with a dose of 200 mcg/kg the following adverse reactions have been reported (frequency): Asthenia/fatigue (0.9%), abdominal pain (0.9%), anorexia (0.9%), constipation (0.9%), diarrhoea (1.8%), nausea (1.8%), vomiting (0.9%), dizziness (2.8%), somnolence (0.9%), vertigo (0.9%), tremor (0.9%), pruritus (2.8%), rash (0.9%), and urticaria (0.9%).

Additionally, in the same population, the following abnormal laboratory tests have been described: elevation in ALT and/or AST (2%) and decrease in leukocyte count (3%). In Participants treated for onchocerciasis with a dose of 100-200 mcg/kg the following adverse reactions have been reported (frequency):

Worsening of *pre-existing*: Arthralgia/synovitis (9.3%), axillary lymph node enlargement and tenderness (11.0% and 4.4%, respectively), cervical lymph node enlargement and tenderness (5.3% and 1.2%, respectively), inguinal lymph node enlargement and tenderness (12.6% and 13.9%, respectively), other lymph node enlargement and tenderness (3.0% and 1.9%, respectively), pruritus (27.5%), skin involvement including oedema, papular and pustular or frank urticarial rash (22.7%), and fever (22.6%).
The following ophthalmological side effects do occur due onchocerciasis itself but have also been reported after treatment with ivermectin: abnormal sensation in the eyes, eyelid oedema, anterior uveitis, conjunctivitis, limbitis, keratitis, and chorioretinitis or choroiditis.

Additionally, the following clinical adverse reactions were reported as possibly, probably, or definitely related to the drug in > 1% of the Participants: facial oedema (1.2%), peripheral oedema (3.2%), orthostatic hypotension (1.1%), and tachycardia (3.5%). Drug-related headache and myalgia occurred in <1% of Participants (0.2% and 0.4%, respectively).

In post-marketing vigilance the following adverse reactions have been reported:

Conjunctival haemorrhage, hypotension (mainly orthostatic hypotension), worsening of bronchial asthma, toxic epidermal necrolysis, Stevens-Johnson syndrome, seizures, hepatitis, elevation of liver enzymes, and elevation of bilirubin.

11. Potential benefits for participants

Participants will not benefit directly from being enrolled in this study. The only benefits for the participants will be information about their general health status and the treatment in the end of the follow up. However, it is hoped that the information gained from this study will contribute

to the development of safe and effective antimalarial drugs. Compensation for missed working day if any and transportation fee for follow-up visit will be reimbursed.

12. Safety assessment

There will be a DSMB set up to assess the safety of the different study dose regimens. Safety of ivermectin treatment will be assessed by analysing the frequency, incidence and nature of adverse events and serious adverse events arising during the study. The respective next treatment arm will only be commenced after the DSMB deems it to be safe.

12.1. Definitions

Adverse Event (AE): An AE can therefore be any unfavourable and unintended sign (including an abnormal laboratory finding), symptom or disease temporally associated with the study, whether or not considered related to the study interventions.

Adverse Reaction (AR): An AR is any untoward or unintended response to ivermectin. This means that a causal relationship between ivermectin and/or *Plasmodium* infection and an AE is at least a reasonable possibility, i.e., the relationship cannot be ruled out. All cases judged by either the reporting medical investigator or the sponsors as having a reasonable suspected causal relationship to ivermectin and/or *Plasmodium* infection will be qualified as adverse reactions.

Unexpected Adverse Reaction: An unexpected adverse reaction is where the nature or severity of the adverse reaction is inconsistent with that expected for the intervention.

Serious Adverse Event (SAE): To ensure no confusion or misunderstanding of the difference between the terms "serious" and "severe", which are not synonymous, the following note of clarification is provided: The term "severe" is often used to describe the intensity (severity) of a specific event (as in mild, moderate, or severe myocardial infarction); the event itself, however, may be of relatively minor medical significance (such as severe headache). This is not the same as "serious," which is based on Participant/event outcome or action criteria usually associated with effects that pose a threat to a participant`s life or functioning. Seriousness (not severity) serves as a guide for defining regulatory reporting obligations.

An SAE is an adverse event that results in any of the following outcomes:

- death;
- a life-threatening adverse event;
- inpatient hospitalization or prolongation of existing hospitalization;

- a persistent or significant disability/incapacity;
- a congenital anomaly/birth defect.

`Life threatening` refers to an adverse effect that at occurrence represented an immediate risk

of death to the participant; it does not refer to an event that hypothetically might have caused death if it were more severe. Similarly, a hospital admission for an elective procedure is not considered a Serious Adverse Event.

Important medical events that may not result in death, be life-threatening, or require hospitalization may be considered a serious adverse event when, based upon appropriate medical judgment, they may jeopardize the participant or require medical or surgical intervention to prevent one of the outcomes listed in this definition. Examples of such medical events include allergic bronchospasm requiring intensive treatment in an emergency room or at home, blood dyscrasias or convulsions that do not result in inpatient hospitalization, or the development of drug dependency or drug abuse.

The following situations do not constitute an SAE : i) hospitalization (including inpatient or outpatient hospitalization for an elective procedure) for a pre-existing condition that has not worsened unexpectedly; ii) hospitalization as a precautionary measure for continued observation or medical management for less than 24 hours, hospitalization for an individual who cannot tolerate oral medication or for hydration); iii) hospitalization to carry out a procedure programmed already before the participant`s participation in the study; iv) hospitalization for social reasons.

Serious Adverse Reaction (SAR): An adverse event (expected or unexpected) that is both serious and, in the opinion of the reporting investigator or sponsors, believed to be possibly, probably or definitely due to ivermectin or any other study treatments, based on the information provided.

Suspected Unexpected Serious Adverse Reactions (SUSARs): A SUSAR is a SAE that is unexpected and thought to be possibly, probably or definitely related to ivermectin.

12.2. Causality assessment

For each AE, an assessment of the relationship of the AE to the study intervention(s) will be undertaken. The relationship of the adverse event with the study procedures will be categorized as unrelated, unlikely to be related, possibly related, probably related, or definitely related. An intervention-related AE refers to an AE for which there is a possible, probable or definite relationship to the study intervention. The investigator will use clinical judgment to determine the

relationship. Alternative causes of the AE, such as the natural history of pre-existing medical conditions, concomitant therapy, other risk factors and the temporal relationship of the event to administration of ivermectin will be considered and investigated.

12.3. Reporting procedures for AEs (Excluding SAEs)

All AEs occurring during the study, since the signature of the Informed Consent Form until the end of the follow up period, observed by the investigator or reported by the participant will be recorded in the CRF. Malaria events occurring at any time during the study will be recorded as an AE.

AEs that result in a participant`s withdrawal from the study or that are present at the end of the study will be followed up (if participant`s consent to this) until a satisfactory resolution or stabilization occurs, or until a non-study related causality is assigned.

Grade 1 Mild: Transient or mild discomfort (< 48 hours); no medical intervention/therapy required;

Grade 2 Moderate: Mild to moderate limitation in activity - some assistance may be needed; no or minimal medical intervention/therapy required;

Grade 3 Severe: Marked limitation in activity, some assistance usually required; medical intervention/therapy required, hospitalization possible;

Grade 4 Potentially life threatening: Extreme limitation in activity, significant assistance required; significant medical intervention/therapy required, hospitalization or hospice care probable.

12.4. Reporting procedures for SAEs

Procedures for Unblinding in case of safety issues

If it is medically imperative to know which trial medication the participant is receiving, the investigator or authorized person should open the randomization envelop. A trial-specific SOP that describes unblinding in detail will be implemented before trial initiation.

Reporting procedure to the Sponsor

Serious Adverse Event (SAE) must be reported within 24 hours by the clinical site to the sponsor, according to sponsor guidelines.

12.5. Reporting Procedures for SUSARS

Investigator will report all SUSARs to the Ethics Committee (EC) within 7 calendar days in case of Fatal and life-threatening SUSARs and within 15 calendar days in case of SUSARs that are not fatal or life threatening.

Investigator will also inform all investigators concerned of relevant information about SUSARs that could adversely affect the safety of participants.

12.6. Safety hold of the study and stopping rules

The study may be placed on safety hold for the following reasons

- On advice of the safety monitor;
- On advice of the investigators;
- On advice of the EC
- One or more participants experience a SAE that is determined to be related to the study product administration;
- One or more grade 3 or higher adverse events which are unexpected and possibly or probably related to the drug administration.

If the study is placed on hold it may only be restarted following discussion with and approval from the Safety monitor, the sponsor and study investigators.

13. Quality control and quality assurance procedures

A safety monitor, i.e. a clinician with experience in clinical trials who is permanently on site and is independent of the clinical trial team, will oversee participant safety.

14. Ethics

This study will be performed in accordance with the current revision of the Declaration of Helsinki and the latest versions of the ICH guidelines for Good Clinical Practice (GCP) and guidelines for Good Clinical Laboratory Practice (GCLP).

14.1. Informed Consent

Written, informed consent will be obtained from all study participants prior to inclusion, as described above. This consent may be withdrawn by the study participant at any time, without being required to provide a reason.

14.2. Ethics review

This trial will be submitted for ethical review to the Institutional Review Board (Comité d'Ethique institutionel, CEI) of the Centre de Recherches Médicales de Lambaréné (CERMEL), which is registered with the Registration Office for Human Research Protections (OHRP) with the numbers: IORG0007336 / IRB00008812.

14.3. Participant Confidentiality

All data will be pseudonymized. Participants' data will be identified by a unique study number in the database. Separate confidential files containing identifiable information will be stored in secured locations. Only the Sponsor representative, investigators, the clinical monitor, and the Institutional Review Board will have access to the records.

15. Data handling and record keeping

15.1. Data Handling

The investigator or his designee will be the data manager with responsibility for delegating receiving, entering, cleaning, querying, analysing and storing all data that accrues from the study. All data will be recorded in case record forms. This includes safety data, laboratory data and outcome data.

15.2. Record Keeping

All files and source documents will be kept confidentially in locked safety cabinets. The investigator, coinvestigators and clinical research nurses will have access to records. The investigators will permit authorized representatives of the sponsor, and the monitors to examine clinical records for the purposes of quality assurance reviews, and evaluation of the study safety and progress.

15.3. Source Data and Case Report Forms (CRFs)

All protocol-required information will be collected in CRFs designed by the investigator. Source documents will be in paper form, and the data will be entered into the clinical trial software. Source documents are original documents, data, and records from which the participant`s CRF data are obtained. For this study these will include, but are not limited to; participant consent form, blood results, laboratory records and correspondence. In this study this will include, but is not limited to medical history, medication records, vital signs, physical

examination records, blood results, adverse event data and details of study interventions. All source data and participant CRFs will be stored securely.

15.4. Data Protection

The study protocol, documentation, data and all other information generated will be held in strict confidence. No information concerning the study, or the data will be released to any un-authorized third party, without prior written approval of the sponsor.

15.5. Procedures for the collection, storage of biological sample

All study research samples are labelled by a code that only the trial site can link to the participant. Samples are stored at the trial site in secure facilities with limited access. Data will be kept in password-protected computers. Only investigators or their designees will have access to the samples and data. Identified samples may be transferred to another facility for analysis if site is not able to analyse the samples for logistical reason. Data will be archived in compliance with national and international guidelines.

16. References

1. Gething PW, Casey DC, Weiss DJ, Bisanzio D, Bhatt S, Cameron E, et al. Mapping Plasmodium falciparum Mortality in Africa between 1990 and 2015. N Engl J Med. 22 2016;375(25):2435-45.

2. WHO | World malaria report 2018 [Internet]. WHO. [cité 30 janv 2019]. Disponible sur: http://www.who.int/malaria/publications/world-malaria-report-2018/en/

3. Sachs J, Malaney P. The economic and social burden of malaria. Nature. 7 févr 2002;415(6872):680-5.

4. TGA eBS - Product and Consumer Medicine Information [Internet]. [cité 30 janv 2019]. Disponible sur: https://www.ebs.tga.gov.au/ebs/picmi/picmirepository.nsf/PICMI?OpenForm&t=&q=Actilyse&r=https://www.ebs.tga.gov.au/

5. Foy BD, Kobylinski KC, da Silva IM, Rasgon JL, Sylla M. Endectocides for malaria control. Trends Parasitol. oct 2011;27(10):423-8.

6. Omura S, Crump A. Ivermectin: panacea for resource-poor communities? Trends Parasitol. sept 2014;30(9):445-55.

7. 2015 Annual Highlights [Internet]. Mectizan Donation Program. 2015 [cité 30 janv 2019]. Disponible sur: https://mectizan.org/news-resources/2015-annual-highlights/

8. Mackenzie CD, Geary TG, Gerlach JA. Possible pathogenic pathways in the adverse clinical events seen following ivermectin administration to onchocerciasis Participants. Filaria J. 24 oct 2003;2 Suppl 1:S5.

9. Kamgno J, Gardon J, Gardon-Wendel N, Demanga-Ngangue null, Duke BOL, Boussinesq M. Adverse systemic reactions to treatment of onchocerciasis with ivermectin at normal and high doses given annually or three-monthly. Trans R Soc Trop Med Hyg. août 2004;98(8):496-504.

10. Njoo FL, Beek WM, Keukens HJ, van Wilgenburg H, Oosting J, Stilma JS, et al. Ivermectin detection in serum of onchocerciasis Participants: relationship to adverse reactions. Am J Trop Med Hyg. janv 1995;52(1):94-7.

11. Ejere HOD, Schwartz E, Wormald R, Evans JR. Ivermectin for onchocercal eye disease (river blindness). Cochrane Database Syst Rev. 15 août 2012;(8):CD002219.

12. Résumé des caractéristiques du produit - STROMECTOL 3 mg, comprimé - Base de données publique des médicaments [Internet]. [cité 30 janv 2019]. Disponible sur: http://base-donnees-publique.medicaments.gouv.fr/affichageDoc.php?specid=61350360&typedoc=R

13. Smit MR, Ochomo E, Aljayyoussi G, Kwambai T, Abong'o B, Bayoh N, et al. Efficacy and Safety of High-Dose Ivermectin for Reducing Malaria Transmission (IVERMAL): Protocol for a Double-Blind, Randomized, Placebo-Controlled, Dose-Finding Trial in Western Kenya. JMIR Res Protoc [Internet]. 17 nov 2016;5(4). Disponible sur: https://www.ncbi.nlm.nih.gov/pmc/articles/PMC5133431/

14. Smit MR, Ochomo EO, Aljayyoussi G, Kwambai TK, Abong'o BO, Chen T, et al. Safety and mosquitocidal efficacy of high-dose ivermectin when co-administered with dihydroartemisinin-piperaquine in Kenyan adults with uncomplicated malaria (IVERMAL): a randomised, double-blind, placebo-controlled trial. Lancet Infect Dis. juin 2018;18(6):615-26.

15. Muñoz J, Ballester MR, Antonijoan RM, Gich I, Rodríguez M, Colli E, et al. Safety and pharmacokinetic profile of fixed-dose ivermectin with an innovative 18mg tablet in healthy adult volunteers. PLoS Negl Trop Dis. 2018;12(1):e0006020.

16. Guzzo CA, Furtek CI, Porras AG, Chen C, Tipping R, Clineschmidt CM, et al. Safety, tolerability, and pharmacokinetics of escalating high doses of ivermectin in healthy adult subjects. J Clin Pharmacol. oct 2002;42(10):1122-33.

17. Prevention C-C for DC and. CDC - Scabies - Resources for Health Professionals - Medications [Internet]. 2018 [cité 30 janv 2019]. Disponible sur: https://www.cdc.gov/parasites/scabies/health_professionals/meds.html

18. Medicines 20 Years Of Helping Australians Make Better Decisions About, Tests M, Technologies OH. Ivermectin (Stromectol) for typical and crusted scabies | RADAR [Internet]. NPS MedicineWise. [cité 30 janv 2019]. Disponible sur: https://www.nps.org.au/radar/articles/ivermectin-stromectol-for-typical-and-crusted-scabies

19. Foy BD, Kobylinski KC, da Silva IM, Rasgon JL, Sylla M. Endectocides for malaria control. Trends Parasitol. oct 2011;27(10):423-8.

20. Chaccour CJ, Kobylinski KC, Bassat Q, Bousema T, Drakeley C, Alonso P, et al. Ivermectin to reduce malaria transmission: a research agenda for a promising new tool for elimination. Malar J. 7 mai 2013;12:153.

21. Alout H, Krajacich BJ, Meyers JI, Grubaugh ND, Brackney DE, Kobylinski KC, et al. Evaluation of ivermectin mass drug administration for malaria transmission control across different West African environments. Malar J. 3 nov 2014;13:417.

22. Kobylinski KC, Alout H, Foy BD, Clements A, Adisakwattana P, Swierczewski BE, et al. Rationale for the coadministration of albendazole and ivermectin to humans for malaria parasite transmission control. Am J Trop Med Hyg. oct 2014;91(4):655-62.

23. Kobylinski KC, Sylla M, Chapman PL, Sarr MD, Foy BD. Ivermectin mass drug administration to humans disrupts malaria parasite transmission in Senegalese villages. Am J Trop Med Hyg. juill 2011;85(1):3-5.

24. Pinilla YT, Lopes SCP, Sampaio VS, Andrade FS, Melo GC, Orfanó AS, et al. Promising approach to reducing Malaria transmission by ivermectin: Sporontocidal effect against Plasmodium vivax in the South American vectors Anopheles aquasalis and Anopheles darlingi. PLoS Negl Trop Dis. févr 2018;12(2):e0006221.

25. Kobylinski KC, Ubalee R, Ponlawat A, Nitatsukprasert C, Phasomkulsolsil S, Wattanakul T, et al. Ivermectin susceptibility and sporontocidal effect in Greater Mekong Subregion Anopheles. Malar J. 07 2017;16(1):280.

26. Kobylinski KC, Foy BD, Richardson JH. Ivermectin inhibits the sporogony of Plasmodium falciparum in Anopheles gambiae. Malar J. 21 nov 2012;11:381.

27. Chaccour C, Lines J, Whitty CJM. Effect of ivermectin on Anopheles gambiae mosquitoes fed on humans: the potential of oral insecticides in malaria control. J Infect Dis. 1 juill 2010;202(1):113-6.

28. Foley DH, Bryan JH, Lawrence GW. The potential of ivermectin to control the malaria vector Anopheles farauti. Trans R Soc Trop Med Hyg. déc 2000;94(6):625-8.

29. Fritz ML, Walker ED, Miller JR. Lethal and sublethal effects of avermectin/milbemycin parasiticides on the African malaria vector, Anopheles arabiensis. J Med Entomol. mars 2012;49(2):326-31.

30. Panchal M, Rawat K, Kumar G, Kibria KM, Singh S, Kalamuddin M, et al. Plasmodium falciparum signal recognition particle components and anti-parasitic effect of ivermectin in blocking nucleo-cytoplasmic shuttling of SRP. Cell Death Dis. janv 2014;5(1):e994.

31. Duthaler U., Suenderhauf C., Karlsson MO. et al. (2018): Population pharmacokinetics of oral ivermectin in venous plasma and dried blood spots in healthy volunteers. Br J Clin Pharmacol. Nov 2018. DOI:10.1111/bcp.1384031.

32. Schoenfeld D. The Asymptotic Properties of Nonparametric Tests for Comparing Survival Distributions. Biometrika. 1981;68(1):316-9.
