# Supplementary material for: Efficacy and safety of ivermectin for the treatment of Plasmodium falciparum infections in asymptomatic male and female Gabonese adults – a pilot randomized, double-blind, placebo-controlled single-centre phase Ib/IIa clinical trial
Source: eBioMedicine. 2023 Oct 13;97:104814. doi: 10.1016/j.ebiom.2023.104814 (PMC10582777; doi:10.1016/j.ebiom.2023.104814)
Supplement: Statistical Analysis Plan [file mmc13.pdf]

Statistical Analysis Plan

**Protocol Title**	Efficacy and safety of Ivermectin for the treatment of *Plasmodium falciparum* infections in asymptomatic Gabonese adults
**Study code**	IVERCURE
**Registration Number**	PACTR201908520097051
**Scope**	Analyses of safety and efficacy of Ivermectin administration in *P. falciparum* infected asymptomatic participants
**Version**	2.2
**Date**	16 January 2023
**Author**	Dorothea Ekoka Mbassi, née Sträßner
**Contributors**	Benjamin Mordmüller, Ghyslain Mombo-Ngoma

Modification history

Version	Date	Author	Modifications
Version 2.2	16 January 2023	Dorothea Ekoka Mbassi, Ghyslain Mombo-Ngoma	editorial changes to study design; endpoints, demographics, efficacy
Version 2	18 January 2021	Dorothea Sträßner, Benjamin Mordmüller	editorial changes, randomization, primary efficacy endpoint, modified intention to treat population, statistical methods: efficacy, conduct of analyses: blinding
Version 1	26 July 2019	Dorothea Sträßner	na

Approved by

	Name	Signature	Date
Author	Dorothea Ekoka Mbassi	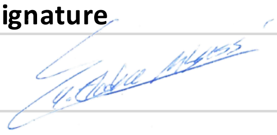	2023-01-16
Study Site Representative	Rella Zoleko Manego		
Sponsor Representative	Ayôla Akim Adegnika		

Statistical Analysis Plan

**Protocol Title**	Efficacy and safety of Ivermectin for the treatment of *Plasmodium falciparum* infections in asymptomatic Gabonese adults
**Study code**	IVERCURE
**Registration Number**	PACTR201908520097051
**Scope**	Analyses of safety and efficacy of Ivermectin administration in *P. falciparum* infected asymptomatic participants
**Version**	2.2
**Date**	09 March 2023
**Author**	Dorothea Ekoka Mbassi, née Sträßner
**Contributors**	Benjamin Mordmüller, Ghyslain Mombo-Ngoma

Modification history

**Version**	**Date**	**Author**	**Modifications**
Version 2.2	14 February 2023	Dorothea Ekoka Mbassi, Ghyslain Mombo-Ngoma	editorial changes to study design; endpoints, demographics, efficacy
Version 2	18 January 2021	Dorothea Sträßner, Benjamin Mordmüller	editorial changes, randomization, primary efficacy endpoint, modified intention to treat population, statistical methods: efficacy, conduct of analyses: blinding
Version 1	26 July 2019	Dorothea Sträßner	na

Approved by

	**Name**	**Signature**	**Date**
Author	Dorothea Ekoka Mbassi		
Study Site Representative	Rella Zoleko Manego	[signature]	09-03-2023
Sponsor Representative	Ayôla Akim Adegnika		

Statistical Analysis Plan

**Protocol Title**	Efficacy and safety of Ivermectin for the treatment of *Plasmodium falciparum* infections in asymptomatic Gabonese adults
**Study code**	IVERCURE
**Registration Number**	PACTR201908520097051
**Scope**	Analyses of safety and efficacy of Ivermectin administration in *P. falciparum* infected asymptomatic participants
**Version**	2.2
**Date**	12 March 2023
**Author**	Dorothea Ekoka Mbassi, née Sträßner
**Contributors**	Benjamin Mordmüller, Ghyslain Mombo-Ngoma

Modification history

Version	Date	Author	Modifications
Version 2.2	14 February 2023	Dorothea Ekoka Mbassi, Ghyslain Mombo-Ngoma	editorial changes to study design; endpoints, demographics, efficacy
Version 2	18 January 2021	Dorothea Sträßner, Benjamin Mordmüller	editorial changes, randomization, primary efficacy endpoint, modified intention to treat population, statistical methods: efficacy, conduct of analyses: blinding
Version 1	26 July 2019	Dorothea Sträßner	na

Approved by

	Name	Signature	Date
Author	Dorothea Ekoka Mbassi		
Study Site Representative	Rella Zoleko Manego		
Sponsor Representative	Ayôla Akim Adegnika	[signature]	12.03.2023

Table of contents

1 Abbreviations 3

2 Study design 4
2.1 Introduction 4
2.2 Design 4
2.3 Sample size 4
2.4 Randomization 4
2.5 Blinding and treatment procedure 5

3 Objectives 5
3.1 Primary objectives 5
3.2 Secondary objective 5
3.3 Exploratory objectives 5

4 Endpoints 6
4.1 Primary efficacy endpoint 6
4.2 Primary safety endpoint 6
4.3 Secondary efficacy endpoints 6
4.4 Secondary safety endpoint 6

5 Study population 6
5.1 Intention to treat population (ITT) 6
5.2 Modified intention to treat population (mITT) 6
5.3 Per protocol population (PPP) 6

6 Data entry 6

7 Statistical methods 6
7.1 Demographics 6
7.2 Safety 6
7.2.1 Grading and causality assessment 7
7.3 Efficacy 7

8 Conduct of analyses 7
8.1 Blinding 7

1 Abbreviations

AE	Adverse Event
ALT	Alanine aminotransferase
AST	Aspartate transaminase
CBC	Complete Blood Count
CRP	C Reactive Protein
DSMB	Data and Safety Monitoring Board
EC	Ethics Committee
GCP	Good Clinical Practice
GGT	Gamma-Glutamyl Transpeptidase
HIV	Human Immunodeficiency Virus
ICH	International Conference on Harmonisation

2 Study design

2.1 Introduction

The Statistical Analysis Plan (SAP) describes the descriptive and inferential statistical analyses of safety and efficacy data during treatment of asymptomatic participants with a *Plasmodium falciparum* infection of 200 to 5000 parasites/µl with 200µg/kg Ivermectine single dose, 200µg/kg for two days, 200µg/kg for three days (dose-escalation stage) or 300µg/kg for three days (randomized-controlled trial). It is a controlled document of the study “Efficacy and safety of Ivermectin for the treatment of *Plasmodium falciparum* infections in asymptomatic Gabonese adults” – IVERCURE. IVERCURE is a single centre trial with two stages. The first stage will be an open label dose escalation trial for safety evaluation while the second stage will conclude the dose escalation and will be conducted double-blinded, randomized and placebo-controlled.

2.2 Design

In total, 49 participants will be recruited in the study. Arms I-III will be conducted sequentially and only Arm IV will be conducted as a randomized controlled assessment.

Dose-escalation stage

- Arm I: 5 participants (200 µg/kg single dose)
- Arm II: 5 participants (2x200 µg/kg)
- Arm III: 5 participants (3x200 µg/kg)

Randomized-controlled trial

- Arm IV:
  a) 17 participants (3x300 µg/kg)
  b) 17 participants (placebo-control)

2.3 Sample size

The first three dose regimens will be tested in five participants. The main aim is to assess safety of ivermectin treatment in the study population in Lambaréné, an endemic region for filarial infections including Loa loa. With this approach there is a 95% probability to detect a relevant Adverse Event occurring at 50% prevalence per arm and cumulatively at a prevalence of 19%. If these doses are shown to be safe, for the final stage there will be 17 participants each for the 3x300 µg/kg regimen and for the placebo-control group with random allocation.
To calculate the sample size of Arm IV, we considered the time to 90% parasite reduction of the participants in the 3-day treatment group versus the time to 90% parasite reduction in the placebo group. Based on previous data, we assume that 25% of volunteers allocated to placebo will reduce parasitaemia to 90% of the initial value within 7 days due to natural acquired immunity and expect that at least 75% of ivermectin-treated volunteers will reduce parasitaemia by 90%. To reach a power of 90%, a single-sided alpha of 2.5% and a ratio 1:1 (treatment versus placebo group), 17 participants per group are required.

2.4 Randomization

At the dose escalation phase, there will be no randomization.
Participants for Arm IV will be randomly allocated to receive either ivermectin or placebo. Randomization will be done by a random number generator implemented in R with the package blockrand using random block sizes between 2 and 3. The randomization ratio will be 1:1 (3-day treatment, control group).

The code to generate the allocation tables is:

blockrand(n = 40, num.levels = 2, levels = c("Placebo", "Ivermectin"), id.prefix = "Arm IV_", block.sizes = 2:3)

A dedicated member of the team, who is not involved in volunteer management or diagnostic activities, will keep the randomization envelopes and dosing schedule. A third party outside the study team and sponsor will generate and distribute the randomization list and randomization cards.
Randomization cards containing the participants arm will be used for randomization in the study and the randomization data are kept strictly confidential until formal interim safety review or the final analysis. At the time of final analysis, the allocation table will be used to calculate outcome and pharmacokinetics.

2.5 Blinding and treatment procedure

A none-blinded person will prepare the treatment assigned by the randomization card, another verifies that the participant's treatment group is assigned by the envelope and complete the corresponding treatment record form. The person administering ivermectin and placebo will be aware of the allocation. Ivermectin and placebo are similar but not identical in appearance and will be administered to each participant separately out of the original packaging without changing the labels. All other study personnel (e.g., physicians, microscopists) and study participants will be unaware of the group allocation. The drugs were administered in a separated room with only one member of the pharmacist team and one participant present at a time, so that neither participants nor other team members would be able to observe the treatment.
The study drugs will be administered under supervision with a fat containing food such as a croissant with bread spread. The two drug administrators (the person administering the drug and the supervising person) are not otherwise involved in the conduct of the trial. The first dose will be administered at the time of the participant's recruitment into the study. Time between blood film preparation and treatment administration will be no longer than four hours. Sub-sequent doses will be given at twenty-four hourly intervals, always together with fat-containing food.

3 Objectives

3.1 Primary objectives

- To assess the safety and tolerability of single- and multiple ascending doses of ivermectin in volunteers with asymptomatic *P. falciparum* infection
- To assess the efficacy of ivermectin in participants with asymptomatic *P. falciparum* infection

3.2 Secondary objective

- To compare efficacy of ivermectin 200µg/kg single dose versus two-day 200µg/kg treatment versus three-day 200µg/kg treatment versus three-day 300µg/kg treatment
- To compare efficacy of ivermectin three-day 300µg/kg treatment compared to placebo
- To compare safety and tolerability of ivermectin 200µg/kg single dose versus two-day 200µg/kg treatment versus three-day 200µg/kg treatment versus three-day 300µg/kg treatment
- To compare safety and tolerability of ivermectin three-day 300µg/kg treatment compared to placebo

3.3 Exploratory objectives

To assess:

- Effect of pharmacokinetic parameters on efficacy on safety
- Activity of ivermectin on *P. falciparum* gametocytes
- Parasite kinetics in the placebo group
- Activity against blood sucking mosquitoes
- Effect on the microbiome

4 Endpoints

4.1 Primary efficacy endpoint

Time to 90% parasite reduction for at least 8 hours assessed by microscopy. This means at least two consecutive thick blood smears show a parasitaemia below 10% of the participant's initial parasitaemia.

4.2 Primary safety endpoint

Number and occurrence of at least possibly related SAE and Grade 3 AE from time of first administration of ivermectin until D7.

4.3 Secondary efficacy endpoints

- Time to 90% parasite reduction assessed by qPCR
- Difference in AUC of parasitaemia until D7
- Parasite clearance time, defined as time to parasitaemia <100 parasites/mL, measured by qPCR

4.4 Secondary safety endpoint

Number and occurrence of any AE from time of first administration of ivermectin until D7.

5 Study population

5.1 Intention to treat population (ITT)

The intention to treat population (ITT) is defined as all randomized participants.
Analysis of the ITT population will be done by allocation to the intervention (e.g., in case of misallocation).

5.2 Modified intention to treat population (mITT)

All participants who received at least one dose of ivermectin.

5.3 Per protocol population (PPP)

All participants who received all doses designated to the allocated group and had a mono-infection with *P. falciparum*, assessed by microscopy and completed follow up until at least day 7. Efficacy will primarily be analysed on the PPP. For sensitivity analysis, the mITT population will be used.

6 Data entry

Data will be entered on paper case report forms (CRF) following procedures outlined in the study protocol and relevant standard operating procedures (SOP). Subsequently, the data from the CRFs are transcribed into an electronic data capture system. For electronic data entry REDCap Version 8.3.1 will be used. One data entry clerk will enter the data which is to be verified by an investigator.

7 Statistical methods

7.1 Demographics

A study flow chart (CONSORT flow chart) will be used to present number of participants screened, enrolled / randomized, treated and followed-up. Distribution of reasons for non-inclusion will be given. Baseline characteristics (age, gender, height, weight, initial parasitaemia) will be tabulated.
Categorical variables will be presented as count and percentage. Numerical variables will be summarized as median and range, parasitaemia as geometric mean and range. For descriptive purposes other aggregation statistics may be done.

7.2 Safety

Adverse events (AE) will be recorded from first dose administration (D0H0) until the end of the trial (D14). Safety data of mITT population will be presented. Adverse events will be tabulated. For AEs that

change in severity, the highest severity will be reported. Verbatim-recorded AEs will be coded using MedDRA and the proportion of subjects with grade 3 AE and SAE classified by MedDRA preferred term level, will be tabulated.

7.2.1 Grading and causality assessment

AE grading – AEs are graded as Grade 1, 2, 3 or 4. Where applicable, using predefined grading schemes. *Relationship to intervention* – The clinical team using five pre-defined levels to assign causality to the study interventions: unrelated, unlikely to be related, possibly related, probably related and definitely related to the intervention.

7.3 Efficacy

The endpoints are:
- Time to 90% parasite reduction for at least 8 hours assessed by microscopy (primary)
- Time to 90% parasite reduction assessed by qPCR (secondary)

The mean time to 10% survival probability of parasites will be analysed with Kaplan-Meier estimator and shown as Kaplan-Meier curve. Differences for intervention and placebo group of the randomized-controlled trial will be calculated by log rank test. Additionally, should the intervention be significantly more efficient than placebo, sex will be added as covariate and data analysed by Cox regression.

- Difference in AUC of parasitaemia until D7

For the randomized-controlled trial, AUC will be calculated by the trapezoidal rule and compared between the ivermectin and placebo arm by Student's t-test.

- Parasite clearance time, defined as time to parasitaemia <100 parasites/mL

The same approach as for 90% will be used. Time until two consecutive parasitaemias <100 parasites/mL will be used as endpoint.

All statistics will be presented with a 95% p-value, as well as confidence interval wher appropriate. The customary $\alpha < 5\%$ will be used as statistical significance threshold.

8 Conduct of analyses

8.1 Blinding

In Arm IV of IVERCURE investigators (clinical and laboratory), participants, data monitors, clinical staff and the sponsor are aware of treatment allocation (blinded). The pharmacy team including the ones administering the drug are unblinded. The data capture system will not contain any information about treatment allocation. Statistical analyses on primary and secondary endpoints will be coded before the blind is lifted using random group assignments.
